# Computational fluid dynamics of the right atrium: Assessment of modelling criteria for the evaluation of dialysis catheters

**DOI:** 10.1371/journal.pone.0247438

**Published:** 2021-02-25

**Authors:** Diana C. de Oliveira, David G. Owen, Shuang Qian, Naomi C. Green, Daniel M. Espino, Duncan E. T. Shepherd

**Affiliations:** Department of Mechanical Engineering, University of Birmingham, Birmingham, United Kingdom; Texas A&M University System, UNITED STATES

## Abstract

Central venous catheters are widely used in haemodialysis therapy, having to respect design requirements for appropriate performance. These are placed within the right atrium (RA); however, there is no prior computational study assessing different catheter designs while mimicking their native environment. Here, a computational fluid dynamics model of the RA, based on realistic geometry and transient physiological boundary conditions, was developed and validated. Symmetric, split and step catheter designs were virtually placed in the RA and their performance was evaluated by: assessing their interaction with the RA haemodynamic environment through prediction of flow vorticity and wall shear stress (WSS) magnitudes (1); and quantifying recirculation and tip shear stress (2). Haemodynamic predictions from our RA model showed good agreement with the literature. Catheter placement in the RA increased average vorticity, which could indicate alterations of normal blood flow, and altered WSS magnitudes and distribution, which could indicate changes in tissue mechanical properties. All designs had recirculation and elevated shear stress values, which can induce platelet activation and subsequently thrombosis. The symmetric design, however, had the lowest associated values (best performance), while step design catheters working in reverse mode were associated with worsened performance. Different tip placements also impacted on catheter performance. Our findings suggest that using a realistically anatomical RA model to study catheter performance and interaction with the haemodynamic environment is crucial, and that care needs to be given to correct tip placement within the RA for improved recirculation percentages and diminished shear stress values.

## Introduction

Haemodialysis is used clinically during kidney failure to support blood filtering. This process is enabled by the use of dialysis catheters, devices with a tip placed within the proximal third of the superior vena cava (SVC), the right atrium (RA), or the inferior vena cava (IVC) [1]. It is extensively used among patients awaiting permanent access placement or maturation [2]. A retrospective study indicated that, in 2011, more than 80% of patients starting haemodialysis in the United States did so through a catheter, where 27% of those undergoing frequent dialysis had a catheter fitted [2, 3]. Several catheter designs are commercially available, differentiated in symmetric, split and step tips, with different features such as the presence or not of side holes [4]. Catheters possess two lumens: the venous lumen brings filtered blood towards the heart while the arterial lumen carries unfiltered blood away from the heart. In addition, they can work in standard or reverse mode, where the latter refers to a switch in venous and arterial lumens. Despite the working mode, all designs must comply with specific requirements: the catheter lumen flow rate must be above 300 ml/min; filtered blood entering the RA needs to be miscible with the blood naturally circulating through the right side of the heart. Therefore, the amount of filtered blood that returns back to the catheter (recirculating flow) should be minimized (below 1%); the tip must not form clots; and catheter segments must not kink [5]. However, catheters have complications such as high rates of infection and dysfunction compared with other forms of dialysis, associated with increased rates of morbidity and mortality in these patients [6, 7]. Different tip designs have been associated with different performances: the step tip is known to have elevated recirculation values in reverse mode, while a symmetric tip catheter is usually associated with lower recirculating flow. This symmetric design is often considered the best design available at present [4, 8, 9].

Both *in vitro* [10, 11] and *in silico* [12–14] studies have assessed catheter performance, to give insight on how to optimise catheter design. *In vitro* studies have used RA models to evaluate catheter performance. An idealized RA model was developed and an *in vitro* set up built for this purpose [10] and an *in vitro* simulator of the RA was employed to study the movement and recirculation associated with different catheter designs [11]. *In silico* studies have used computational fluid dynamics (CFD) to study blood flow patterns associated with different catheter designs, as well as evaluating recirculation and thrombosis. Such studies have included the comparison of symmetric catheters [13], the preclinical assessment of novel designs [12] and the evaluation of different tip hole shapes [14]; however, none included the use of a geometry representing the RA.

While left heart function has been extensively explored using computational modelling [15–18], the right side has been mostly neglected. Only a few studies have focused on the RA [19, 20], with only one CFD study employing the use of a realistic RA geometry to study the performance of a single catheter design [20]. The use of a realistic RA computational model for evaluation of catheter performance is, however, a current need, since this allows a more accurate representation of the *in vivo* behaviour experienced by dialysis catheters due to the surrounding physiological flow patterns and allows for a less costly and faster prediction of the subsequent haemodynamics without the need for an *in vitro* test setup.

The aim of this study was to develop a physiological CFD model of the RA which enables the assessment of the performance of a range of designs for dialysis catheters. The CFD model was validated against data in the literature and then used to evaluate the performance of four different catheter designs, including one split tip, one symmetric tip and two step tips (with and without the presence of side-holes). For the step designs, reverse flow mode was employed, and the haemodynamic features evaluated include flow recirculation and the assessment of shear stresses in blood.

## Methods

### Domain description and discretization

**Fig 1:**
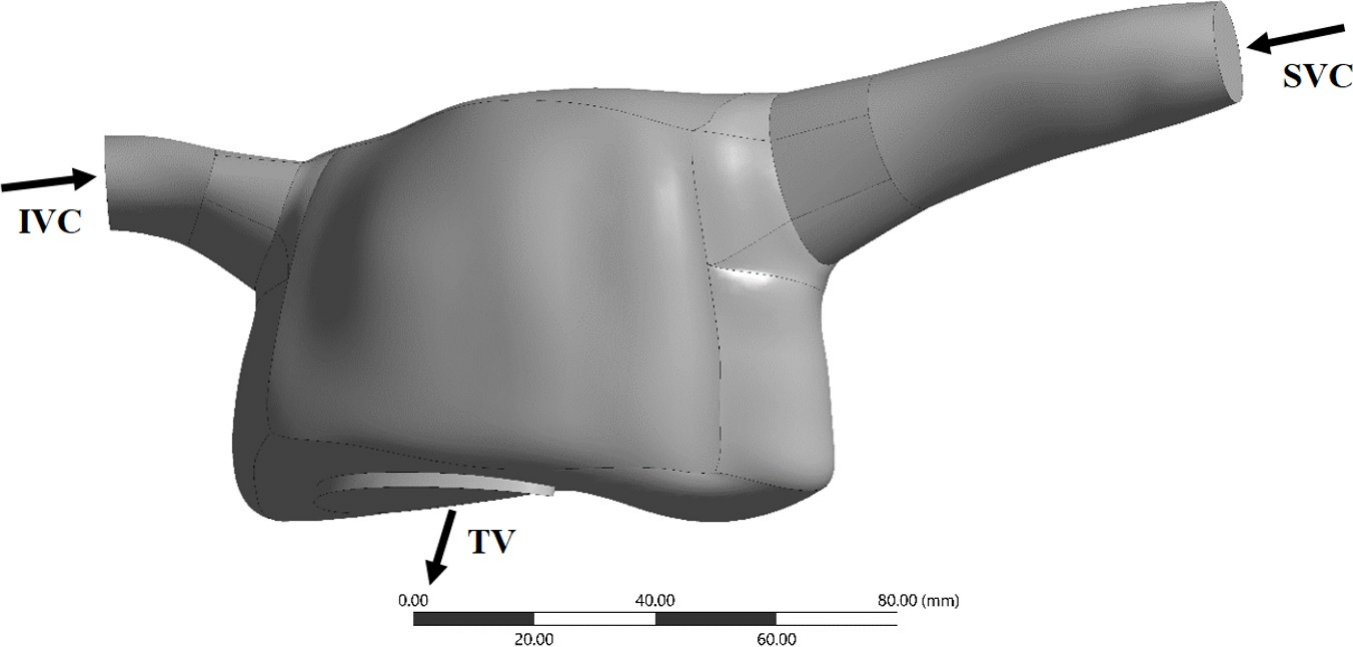
RA computational domain. IVC, inferior vena cava; SVC, superior vena cava; TV, tricuspid valve.

A 3D reconstructed RA geometry was retrieved from a healthy human heart model present in GrabCAD; this model was built from data in the literature using SolidWorks (https://grabcad.com/library/the-human-heart-1). The model was truncated at the superior vena cava (SVC), inferior vena cava (IVC) (inlets) and tricuspid valve (TV) (outlet), The geometry was re-scaled to match IVC and SVC physiological mean literature diameters using ANSYS SpaceClaim v.18.2 (Ansys Inc., Canonsburg, PA, USA). Final diameters for the IVC and SVC yielded 16.89 mm and 17.06 mm, respectively, and the area of the TV was 9.95 cm^2^, within *in vivo* ranges [21–24]. Face and edge repair tools from ANSYS SpaceClaim were then used to repair the RA model surfaces, yielding the geometry presented in Fig 1. It is noted that there is an elevated variability in the data reported in literature for the dimensions of the RA, which may be due to variability in subject populations, image acquisition methods and subsequent empirical determination of right atrial volumes [25]. Nonetheless, the volume of our RA geometry, excluding the cava veins, was 175.27 mL, slightly higher than values reported for healthy subjects (upper limit of normality: 170.4 mL [26]), but still within ranges presented by previous clinical papers [25, 26].

**Fig 2:**
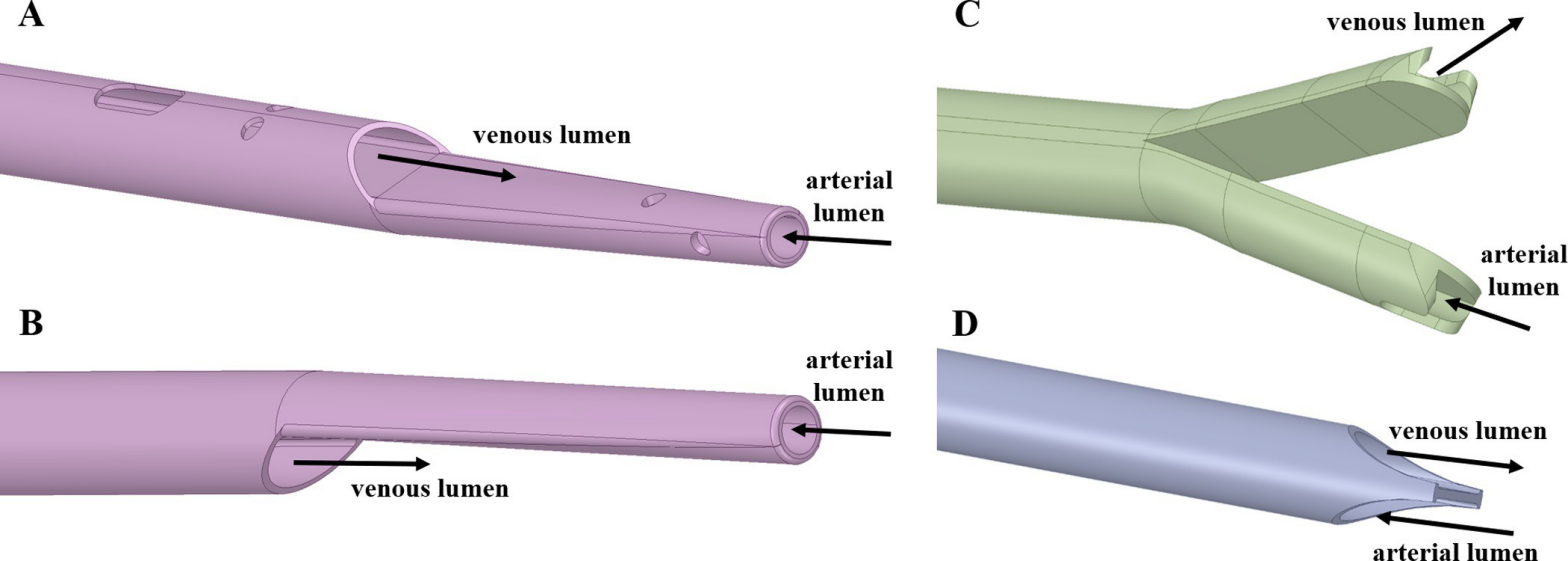
Catheter tip designs A, B, C and D, with arterial and venous lumens indicated. Catheters A and B were set in reverse mode (for C and D designs, forward and reverse mode lead to the same model configuration).

**Fig 3:**
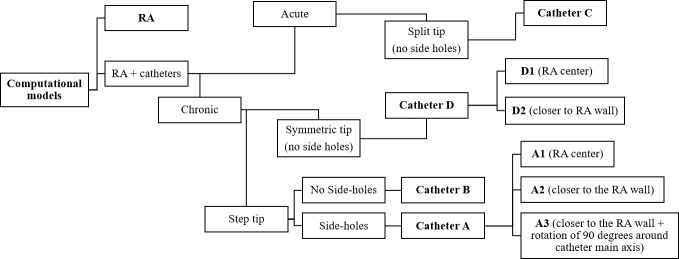
Diagrammatic overview of all computational models developed. Acute refers to temporary catheter placement and chronic to permanent catheter placement, this is of relevance to the clinical use of the catheters.

Four catheter designs were chosen: catheters A and B have a step tip with and without side-holes, respectively. Catheter C has a split tip and catheter D has a symmetric tip, without side holes (see Table 1 for dimensions). The geometries of the four catheters are shown in Fig 2 (S1–S4 Files). A total of 8 computational models (including the RA model) were designed, as outlined in Fig 3.

**Table 1:** Catheter dimensions.

Catheter name	Tip length [mm]	Outer diameter [Fr]	Lumen area [mm^2^]
A	290	15.5	7.8
B	290	15.5	7.8
C	152	15	3.5
D	240	16	7.8

Notes: Fr, French gauge; 1 Fr = 0.33 mm.

**Fig 4:**
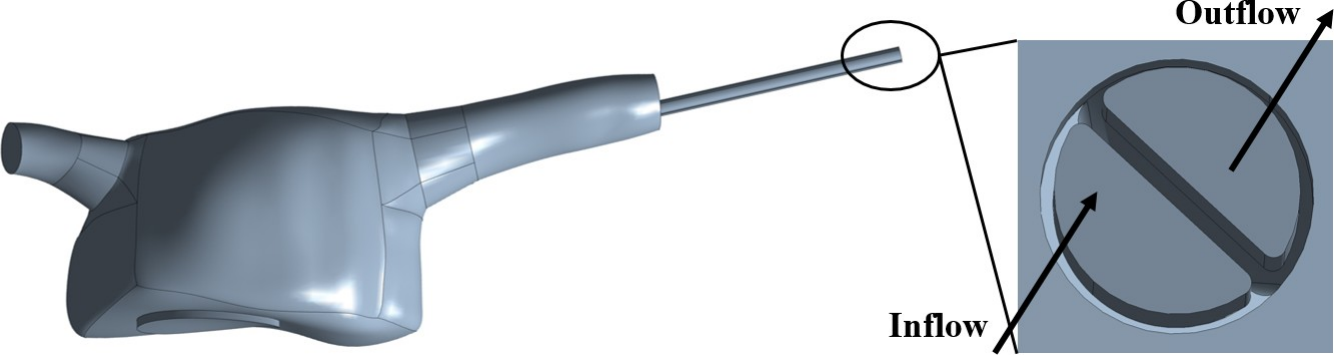
RA computational domain with example catheter inserted.

3D geometry files corresponding to catheters designs A, B, C and D were placed in the RA geometry. Catheters were placed through the SVC using ANSYS SpaceClaim, with their entire functional part inside the RA and their venous tip placed well past the SVC in the central region of the RA, to mimic clinical guidelines [27]. For each catheter model, the structure was removed and only a unified volume including the fluid within the RA and the fluid within the catheter structure was used for subsequent CFD simulations. An example of an inserted catheter into the RA can be seen in Fig 4.

**Fig 5:**
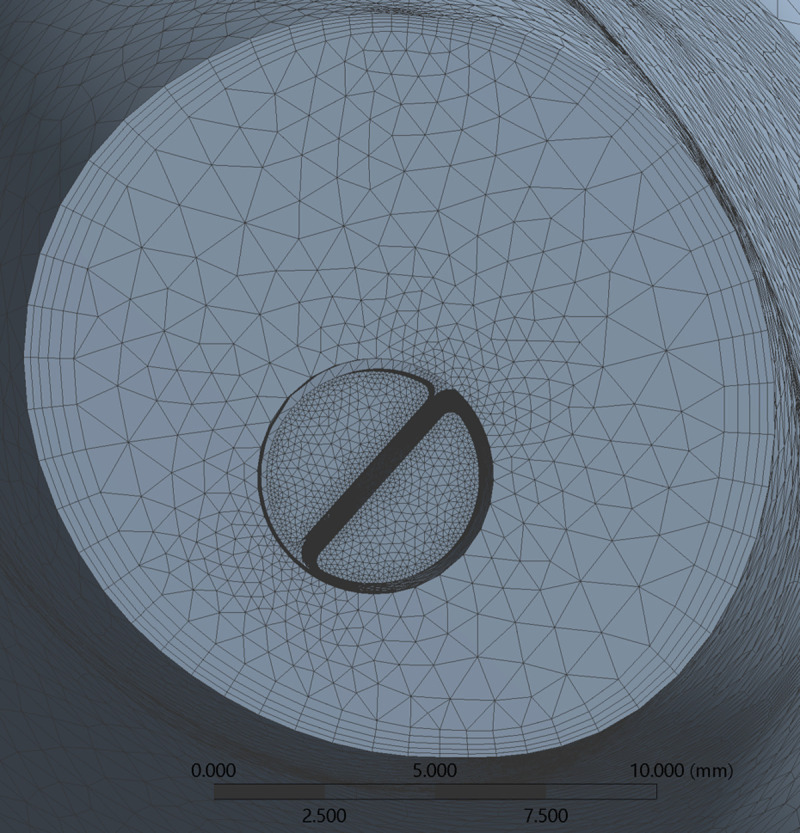
Finite-element mesh cross-section of catheter B inserted in RA.

All geometries were meshed using ANSYS (Ansys Inc., Canonsburg, PA, USA) and an example of a catheter model mesh within the RA is provided in Fig 5. Tetrahedral elements were employed, with a patch conforming method scheme. This scheme allowed the choice of a finer mesh for catheter fluid boundaries in order to achieve greater mesh refinement within this volume. A structured hexahedral mesh was used for the creation of 5 boundary layers in the fluid near the right atrial wall. Using these settings, an average spatial resolution of 0.1 mm was achieved for the solid mesh inside each catheter while 1 mm was achieved for the remaining solid mesh, respectively. Mesh quality was assessed through element skewness and orthogonal quality (Table 2). According to quality criteria, our meshes had excellent skewness features (between 0 and 0.25) and very good orthogonal quality (between 0.70 and 0.95) [28, 29].

**Table 2:** Mesh settings and quality assessment.

Model	No. mesh elements	Average orthogonal quality	Average skewness
RA	1,144,719	0.797	0.200
A1	3,325,107	0.773	0.226
A2	3,316,264	0.773	0.226
A3	3,342,044	0.773	0.226
B	3,294,202	0.773	0.226
C	3,460,285	0.787	0.212
D1	3,236,187	0.770	0.228
D2	3,237,906	0.772	0.227

**Fig 6:**
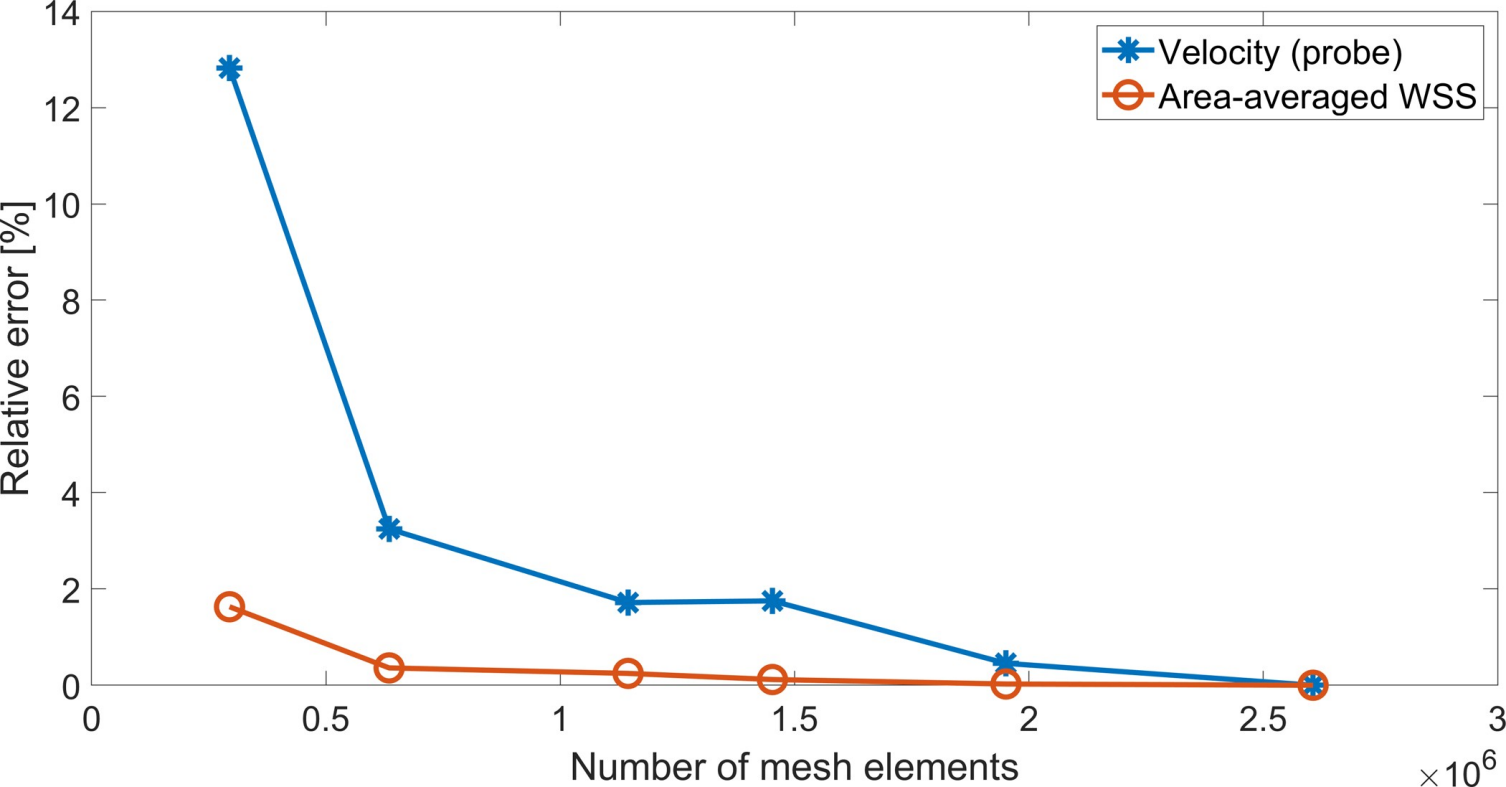
Mesh convergence study for RA model.

A mesh convergence analysis was performed using the RA model, with mesh refinement being achieved by progressively decreasing tetrahedral element size in the ANSYS Meshing Module. The mesh convergence test was performed using ANSYS Fluent 18.2 by running one cardiac cycle. For all meshes, the instantaneous velocity magnitude at a probe located at the centre of the RA (S5 File) and the surface averaged WSS were obtained. The relative error between values obtained under increasing mesh density and the solution obtained when using the finest mesh was then evaluated (S6 File and Fig 6). Given the complex flow patterns developing inside the RA, we assumed an error below 0.5% as acceptable for the average WSS and below 3% for the probe velocity. Both criteria were met once the model had above 1 million elements.

### Flow governing equations and material properties

Given the importance of using non-Newtonian models for the study of local haemodynamics [30], and similarly to the recent paper by Owen et al. (2020) [31], blood was considered a Non-Newtonian fluid [32] and modelled using the Bird-Carreau model:

$$\mu =\mu_{\infty }+(\mu_{0}-\mu_{\infty }){\left[1+{\left(\lambda \dot{\gamma }\right)}^{2}\right]}^{(n-1)/2}$$ (1)

where *μ* is the blood viscosity, *μ*_∞_ is the high shear viscosity, *μ*_0_ is the low shear viscosity, *λ* is the time constant, $\dot{\gamma }$ is the shear rate and *n* is the Power law index [33]. As per previous studies, the following values were used: *λ* = 3.313 s, *n* = 0.3568, *μ*_0_ = 0.056 Pa s and *μ*_∞_ = 0.00345 Pa s [33, 34] and a blood density of 1060 kg/m^3^. A comparison between this non-Newtonian model and a Newtonian model, employing the same blood density and a blood viscosity of 0.004 Pa s, is provided for the RA model in the S7 File.

The Reynolds number (Re) obtained from the equation for a haemodynamic chamber [35] was evaluated for each boundary of the RA model (SVC, IVC and TV). The Reynolds number is defined as

Re=ρUDμ (2)

where ρ is the blood density, **U** and *μ* are the velocity magnitude and viscosity at each boundary, and D is the diameter of the corresponding boundary. Averaged Reynolds numbers have been determined based on time and space-averaged values of **U** and *μ* over each boundary, while the minimum and maximum Reynolds values were computed for a time-varying Reynolds number obtained using the averaged **U** and *μ* over each boundary (Table 3). Since Re << 2300, flow was assumed to be laminar [35].

**Table 3:** Reynolds number study performed for the RA model.

Boundary	Minimum Reynolds	Mean Reynolds	Maximum Reynolds
SVC	837	1120	1270
IVC	684	1170	1400
TV	760	1140	1390

### Boundary conditions

#### RA model

**Fig 7:**
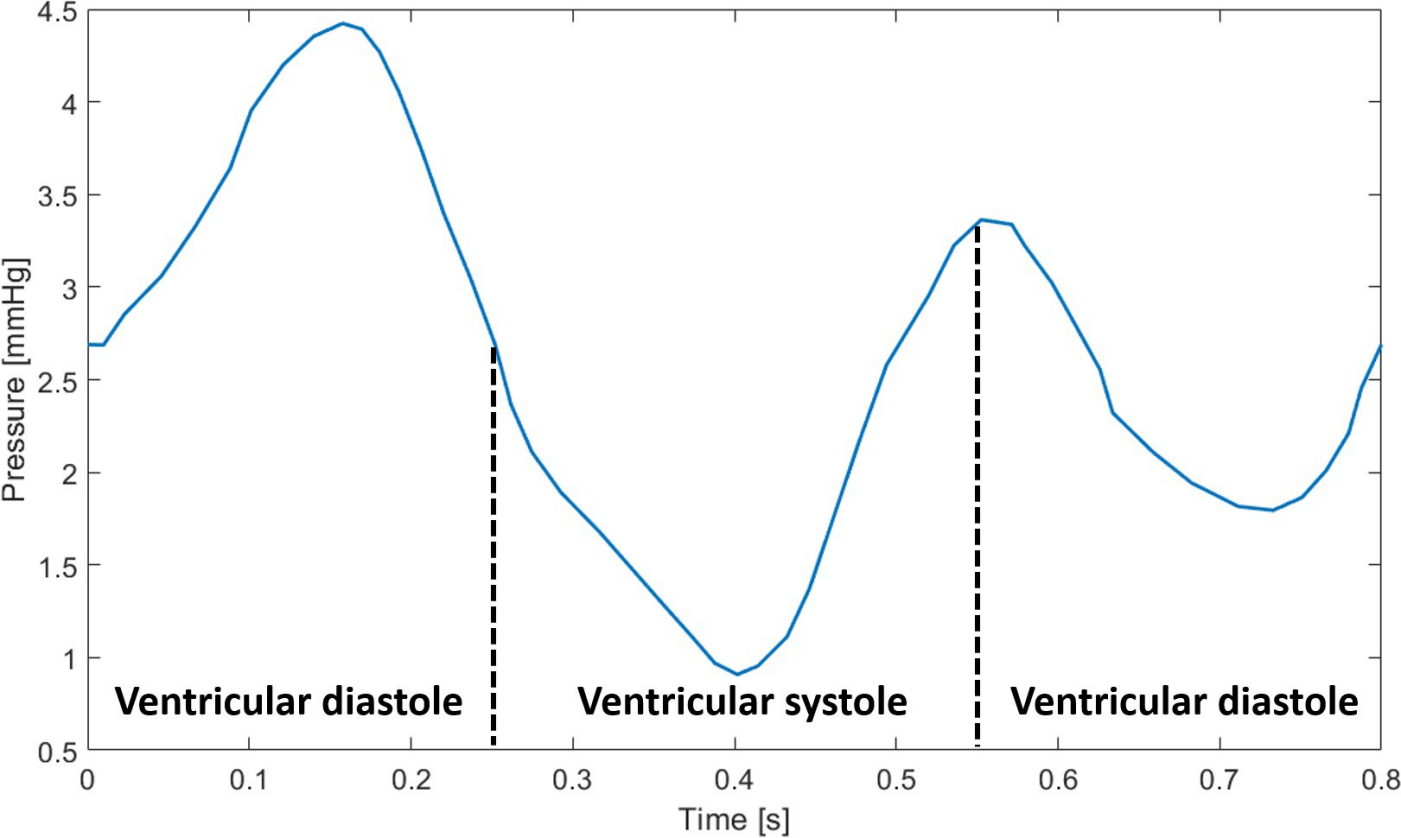
Time-dependent pressure, with diastolic and systolic periods represented, imposed at the inlets (adapted from Cohen *et al*. 1986). This boundary condition can be generated with the code from the S8 File.

To accurately represent the pulsatile behaviour of blood flow, a time-dependent physiological pressure two-dimensional waveform was applied at the SVC and IVC inlets (Fig 7) [36], modelled as a spatially uniform profile. According to this waveform, the complete cardiac cycle corresponds to a period of 0.8 s, and the diastolic and systolic periods last for 0.5 s and 0.3 s, respectively. The TV outlet was set to a constant gauge pressure of 0 Pa. The right atrial wall boundaries were assumed rigid and a no-slip condition was employed at the wall-blood interface.

#### Catheter models

Catheter tip designs employed in our study are shown in Fig 2. The venous and arterial lumens correspond to the placement of inlet and outlet boundary conditions, respectively. For all models with catheters inserted into the RA, the boundary conditions applied for the RA model (and represented in Fig 1) were also included. Although the pressure (and therefore the respective flow rate) generated by a dialysis machine oscillates [5], blood flow through the catheter inlet (venous lumen) was set to a constant volume flow rate of 400 ml/min (the maximum flow rate used clinically), together with a constant gauge pressure of 248 mmHg [5]. At each catheter outlet (arterial lumen), different constant gauge pressures were applied to represent a flow rate within the clinical range, as specified in Table 4.

**Table 4:** Gauge pressure values applied at catheter outlets and respective flow rates achieved [5].

	A	B	C	D
**Pressure [mmHg]**	-250	-250	-250	-188
**Flow rate [ml/min]**	350	380	360	370

### Computational settings

ANSYS Fluent 18.2 (Ansys Inc., Canonsburg, PA, USA) was used to implement and solve the CFD simulations, with fluid dynamics being solved using the continuity and incompressible Navier-Stokes equations [37] under transient conditions. While a single-phase model was assumed for the RA model alone, a multiphase model was set up for all catheter geometries. This choice was made to allow for the quantification of recirculating flow. Two phases simulating blood with identical material properties were defined, where the initial volume of flow entering the RA through the catheter was assumed as one phase (recirculation phase–filtered blood), and the remaining flow was assumed as the other (primary phase–unfiltered blood) [38]. Through this process, the flow passing through the venous lumen and the volume of recirculation phase flow entering the arterial lumen of the catheter were monitored and quantifiable. Further details of simulation set-up are summarized in Table 5.

**Table 5:** CFD set-up.

Type	Choice	Description
Solver for continuity equation	SIMPLE [38]	Coupled pressure-velocity
		Segregated approach
		Second order upwind momentum
Solver for pressure discretization	PRESTO!	
Transient solving	First order implicit	Solving of each time step iteratively
Multiphase general settings	Two Eulerian phases with equal material properties	Primary phase–fluid entering/exiting all boundaries except catheter inlet
		Recirculation phase–fluid entering catheter inlet
Multiphase volume fraction [38]	Explicit formulation	
	Sharp/dispersed interface	
	Implicit body force	
Time stepping	Single phase: 0.005 s	
	Multiphase: 0.001 s	
Convergence criteria	Residual error < 10^−4^	Residual errors for continuity and x-, y-, and z- velocities

To determine how many cycles were necessary to achieve temporal convergence, the RA model was run for 8 cycles. The time-averaged mean velocity and pressure were analysed for each cardiac cycle. The relative error (*E*) between the approximate velocity and pressure solutions for each cycle and the solution from the last cycle (7^th^ cycle) was then measured, using Eq 3:

$$\text{E}=\left|\frac{v_{B}-v_{L}}{v_{L}}\right|\cdot 100\%,$$ (3)

where *v*_*B*_ and *v*_*L*_ are the calculated variables for each time period before the last and for the last time period, respectively.

**Fig 8:**
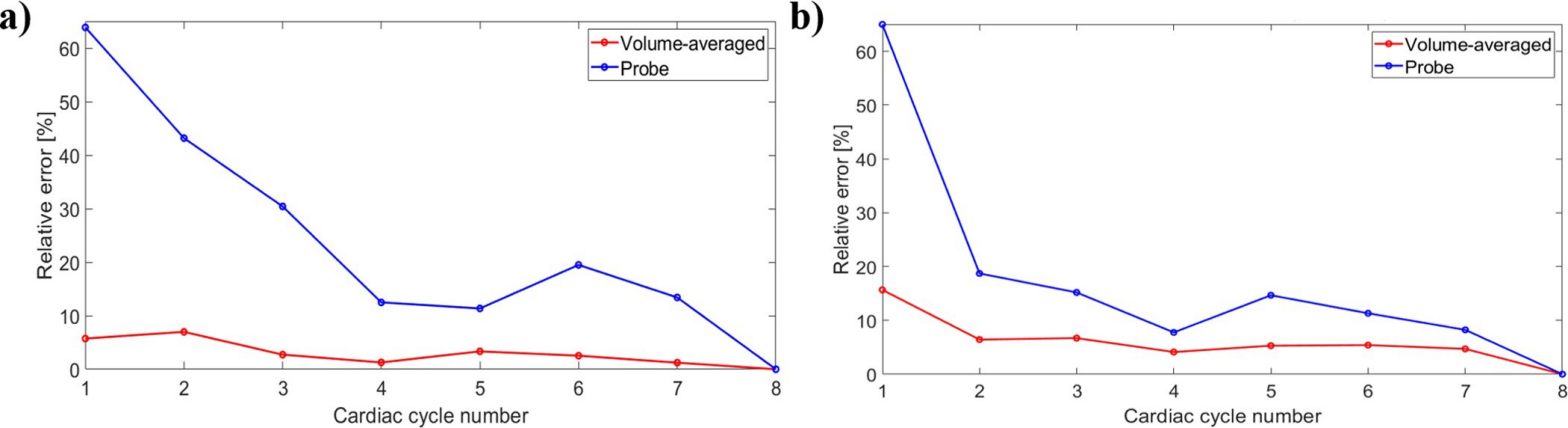
Relative percentage error for velocity (a) and pressure (b) measurements with increasing number of cardiac cycles.

Eq 3 was employed for volume-averaged derived velocity and pressure and for probe related quantities (S9 File), with the probe placed at the centre of the RA. A decrease in the error was observed with increasing number of cycles for volume-averaged and probe velocity and pressure (Fig 8): here, the last data point is at the 7^th^ cycle and it is noted that volume-averaged results have an associated relative error smaller than those of localised velocities and pressure, which are less prone to stabilize. The 4^th^ cardiac cycle was chosen for result retrieval and quantifications, corresponding to relative errors of 1.28% and 4.12% for volume-averaged velocity and pressure quantities, respectively.

All simulations were run for a total of 3200 time steps, corresponding to 3.2 s (4 cardiac cycles), with the results being retrieved from the last cycle. All numerical simulations were performed using super-computing facilities (BlueBear High Performance Computing, University of Birmingham), with 100 cores for each simulation and 5 GB RAM per core. The solution time for the simulation of the RA model was ~10 hours, and ~25 hours for the models of the RA with a catheter included.

### Haemodynamics

All simulation results were accessed using Ansys CFD-post. Global right atrial haemodynamics were evaluated in terms of velocity magnitude, flow streamlines, vorticity, pressure and wall shear stress (WSS, both time-averaged and non-time-averaged). Vorticity **(ω**) is defined as the curl of the velocity field [39], and describes the local spinning motion of blood flow, as follows

ω=∇×u (4)

where **u** is the velocity vector. WSS was calculated and averaged over the whole RA wall. Time-averaged quantities were also derived considering the cardiac cycle period (0.8 s). All RA results were then validated against the available literature data before using it as a haemodynamic model for catheter performance studies (section 3.1).

Catheter performance was evaluated using measures of vorticity, WSS, recirculation and blood shear stress. To calculate recirculation of blood through catheters, the volume fraction (0–1) of the recirculation phase (∅_r_) is considered: for multiphase catheter models, any measure in a mesh element is weighted between the primary and the recirculation phases. The time-averaged volume fraction of the recirculation phase at the catheter outlet is then defined as

$$\bar{\emptyset_{\text{r}}}=\frac{1}{\text{T}}\int_{0}^{\text{T}}\emptyset_{\text{r}}\text{dt}$$ (5)

where T is the length of the cardiac cycle. For a k^th^ facet of the catheter outlet boundary, the magnitude of the recirculation fraction can then be defined as a mass-weighted average of $\bar{\emptyset_{k}}$ through the catheter outlet,

$${\text{R}}_{\text{f}}=\frac{1}{{\dot{\text{m}}}_{\text{T}}}\sum_{\text{k}=1}^{n}\bar{\emptyset_{\text{k}}}{\dot{\text{m}}}_{\text{k}}$$ (6)

where $\dot{m_{T}}$ and $\dot{m_{k}}$ are the total mass flow rate over the catheter outlet boundary and the mass flow rate for a k^th^ facet, respectively. The latter is defined by

$${\dot{\text{m}}}_{\text{k}}=\rho (u_{\text{k}}\cdot A_{\text{k}})$$ (7)

where **u**_k_ and **A**_k_ are the k^th^ facet velocity and area vectors, respectively [40].

Previous studies have noted higher levels of platelet activation at the venous lumen tip in comparison with the arterial one, with the latter yielding small differences amongst catheter designs [13]. To better observe any marked differences between models, we chose the venous tip for analysis and quantification of shear stress, which can be defined as

$$\tau =\mu \cdot \left|\epsilon_{ij}\right|,$$ (8)

where τ is the shear stress and $\left|\epsilon_{ij}\right|$ is the magnitude of the strain rate. The strain rate tensor and its magnitude are defined by Eqs (9) and (10), respectively,

$$\epsilon_{ij}=\frac{1}{2}\left(\frac{\partial {\text{u}}_{\text{j}}}{\partial {\text{x}}_{\text{i}}}+\frac{\partial {\text{u}}_{\text{i}}}{\partial {\text{x}}_{\text{j}}}\right),$$ (9)

$$\left|\epsilon_{ij}\right|=\sqrt{2\epsilon_{ij}\epsilon_{ij},}$$ (10)

where u_i_ and u_j_ are the velocity vectors in the i and j directions and x_i_ and x_j_ are the spatial coordinates in the i and j directions [40].

**Fig 9:**
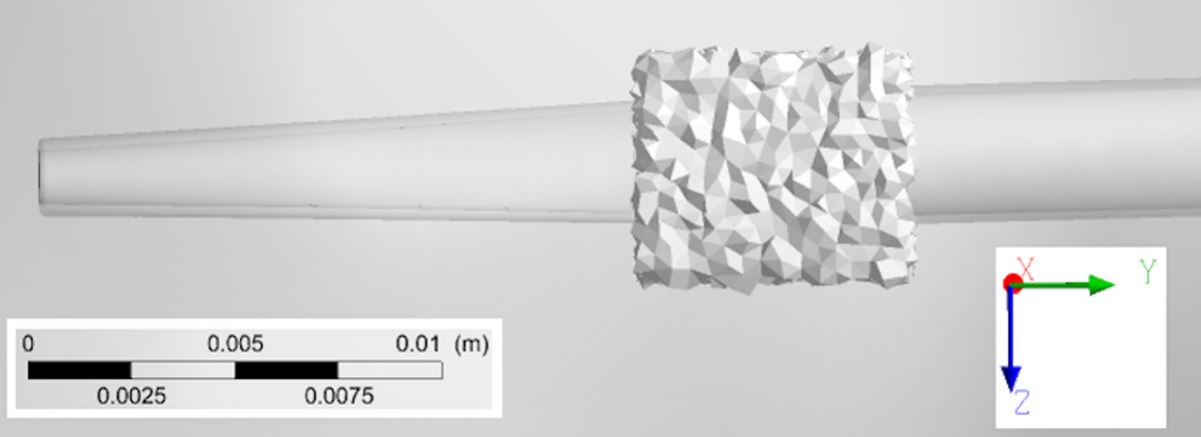
Example of volume definition to be placed at the tip.

Furthermore, rectangular prism volumes were created at each venous tip [14] by defining (x, y, z) boundaries in ANSYS CFD-post (example of volume definition in Fig 9). The size of all tip volumes is specified in Table 6. For catheter A, the tip volume included all orifices of the tip.

**Table 6:** Dimensions for the creation of tip volumes for all catheters.

	A	B	C	D
**Length [mm]**	4.5	8	7	13
**Width [mm]**	25	10	7	9
**Height [mm]**	6	6	7	5.5

At this tip volume, volume-averaged shear stress was calculated, according to:

$$\bar{\tau }=\frac{1}{\text{V}}\int {}_{tip}\tau \;\text{dV},$$ (11)

where V is the tip volume. The tip volume (%) where τ ≥ 10 Pa was evaluated to identify the potential for platelet activation [14, 41].

## Results

### Right atrium model validation

Right atrial CFD results were compared against *in vivo* and *in vitro* results from the literature; this comparison focused on data for a healthy RA model without the presence of a catheter. This approach to validation meant that more data was available from literature for comparison. Blood velocity magnitudes were within the range of those presented by *in vivo* reports. The time- and volume-averaged velocity magnitude was 0.192 m/s, while the minimum and maximum values of the volume-averaged velocity magnitude oscillated between 0.160 m/s and 0.233 m/s, respectively. The average value was in the range of reported values by previous clinical (0.174 ± 0.027 m/s) [42] and *in vitro* [43] studies. In addition, the predicted time- and volume-averaged pressure was 1.18 mmHg, while the minimum and maximum values of the volume-averaged pressure varied between 0.55 and 1.88 mmHg, respectively. Maximum pressure values reached 4.55 mmHg. Clinical guidelines specify that, for an IVC diameter < 21 mm (as is the case of the model), the normal time- and volume-average RA pressure is 3 mmHg, with minimum and maximum volume-averaged values varying between 0 and 5 mmHg [44]. Therefore, the obtained computational pressure is within estimated clinical values.

**Fig 10:**
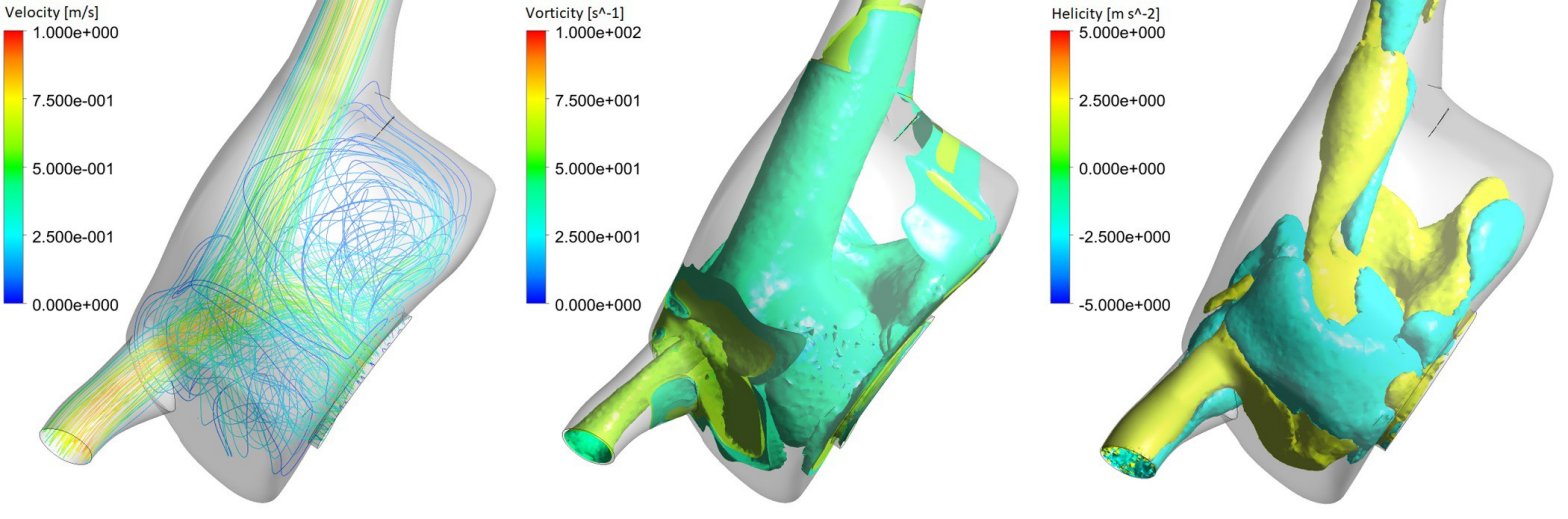
Right atrial flow patterns: Streamline fields representing velocity magnitude are presented (left), as well as isosurfaces representing vorticity (middle) and helicity (right), at the beginning of systole (t = 0.25 s).

The model also predicted characteristic flow patterns within the RA, with the presence of a vortex originated from the IVC flow and SVC flow swirling around it in a helical fashion (Fig 10 –left). This is also corroborated by clinical studies, which speculate that this swirling motion optimises blood flow within the heart [42, 43, 45, 46]. The predicted volume-averaged vorticity for the RA was 44.1 s^-1^, which is within the range of *in vivo* predictions (37–54 s^-1^) [45]. Fig 10 also shows the presence of both vortical and helical features, with counter-rotating flow filling the RA (positive and negative helical structures). Although a purely clockwise vortex is most common [47, 48], a spectrum of right atrial flow patterns exists in the structurally normal heart, including vortical, helical and multiple vortical flow [42], consistent with the predicted flow patterns.

**Fig 11:**
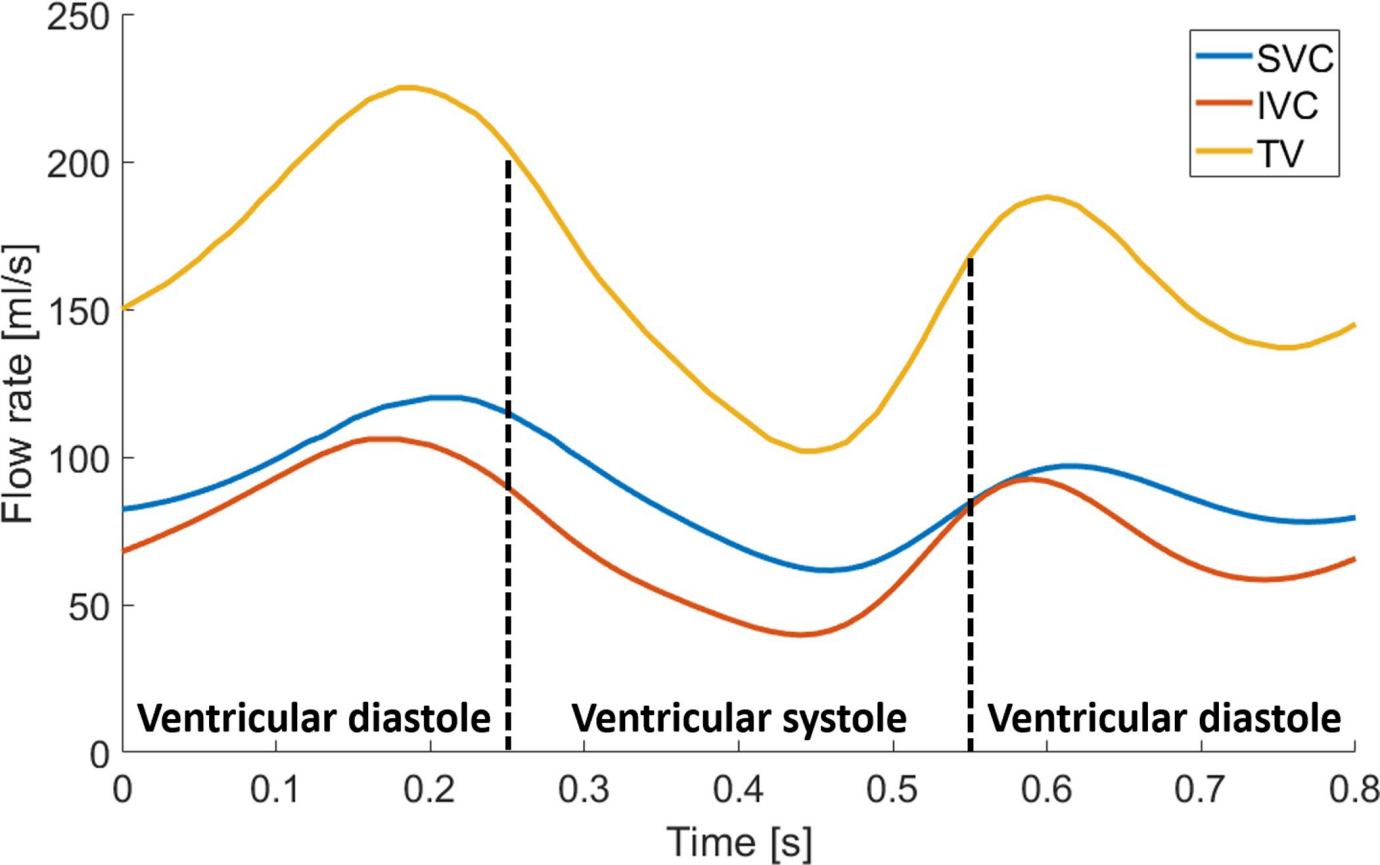
Blood flow rate profiles over one cardiac cycle at SVC, IVC and TV boundaries (generated with data from the S10 File).

The time-evolution of flow rates through the SVC, IVC and TV over a cardiac cycle period are presented in Fig 11. Similar to the literature, both SVC and IVC have similar flow rate waveforms, with a two-peaked shape (a peak at ventricular systole and a smaller one at ventricular diastole) [42, 49]. Literature shows that the range of these flow rate waveforms can greatly vary amongst a population sample and with age [49]. Our predictions yield maximum systolic flow rate values of 106 ml/s and 120 ml/s for the IVC and SVC. While the obtained waveform and maximum for the IVC are consistent with clinical predictions (maximum value of 151.1 ± 55.3 ml/s at systole for a group of healthy adults aged 20–39 years), the SVC waveform and maximum value are overestimated (maximum value increased by 22% in comparison with the maximum standard deviation observed in the literature [49]).

TV function was not included in the model (see Limitations section); hence, the TV flow rate waveform follows a similar pattern in comparison with that of the IVC and SVC, with a peak flow rate of 225 ml/s. However, the current literature does not present many examples of the expected flow rate through the TV making any comparison difficult. One study shows that there is a phase shift in the location of the TV flow rate peaks, which should appear during the ventricular diastolic period [50]. This did not occur in our flow rate predictions, likely because TV function was not modelled.

The haemodynamic results obtained for the RA, including WSS and vorticity, are further discussed in Section 3.2, as well as compared with those obtained by our CFD catheter models.

### Catheter insertion

Recirculation, vorticity, time-averaged WSS magnitude and shear stress results are presented in Table 7 for the RA model and all catheter designs.

**Table 7:** Haemodynamic predictions for all catheter models.

Quantity	RA	A1	A2	A3	B	C	D1	D2
R_f_ [%]	-	9.32	6.45	8.84	43.7	9.71	0.26	0.21
Time and volume-averaged ω [s^-1^]	44.10	54	55.20	55	57.20	56.10	55.80	54.90
Time-averaged WSS [Pa]	1.89	2.01	2.04	2.05	1.93	1.97	2.15	2.14
Time-averaged $\bar{\tau }$ [Pa]	-	12.90	15.50	13.70	13.80	11.20	11.60	10.20
Vol. time-averaged τ > 10 Pa [%]	-	28.30	33.40	28.60	28.10	15.70	28.70	28.50

Notes: Recirculation, vorticity and shear stress are time-averaged over one cardiac cycle.

#### Impact of catheter insertion on RA haemodynamics

**Fig 12:**
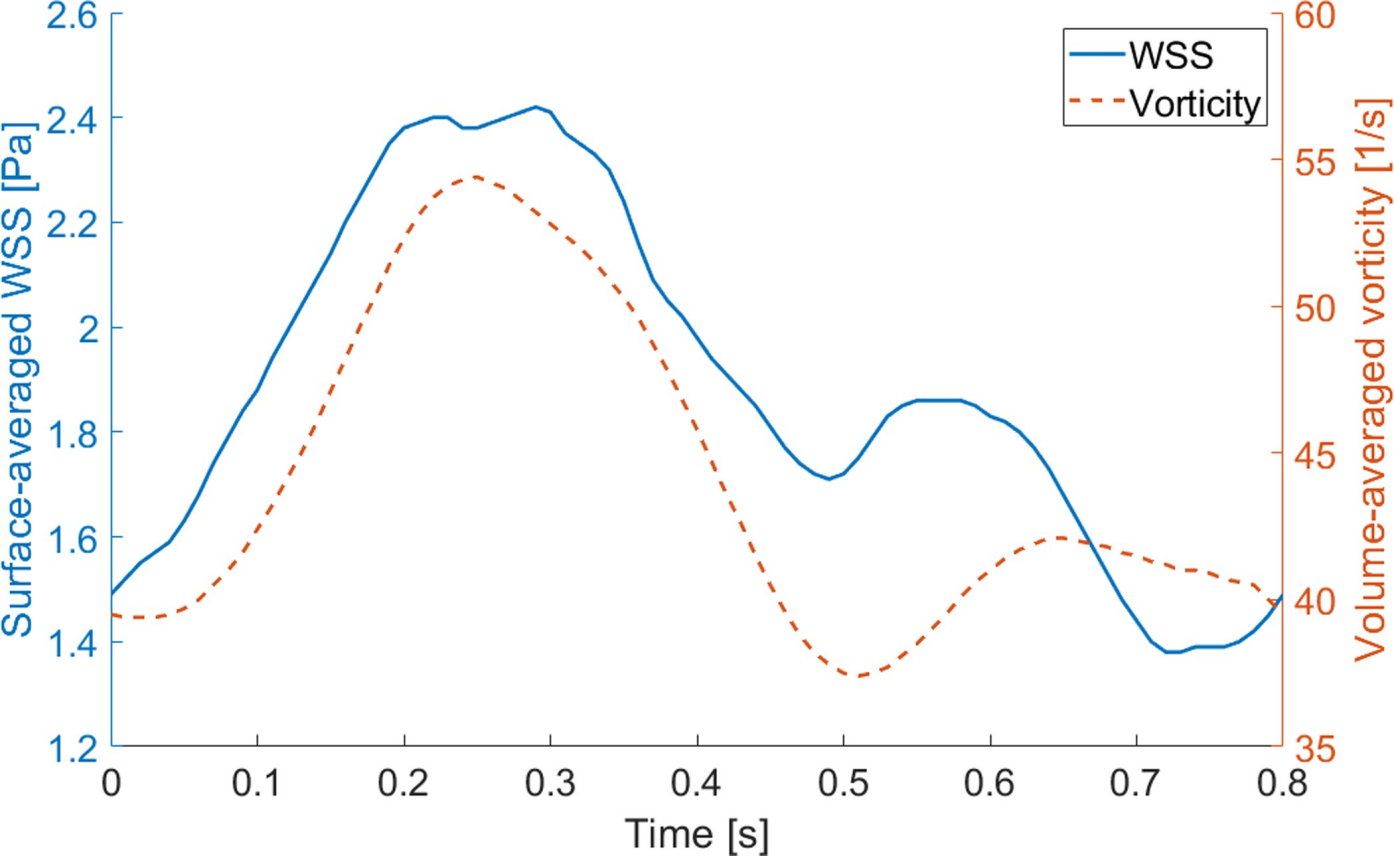
Time evolution of spatially averaged WSS and volume-averaged vorticity (generated with data from S11 File).

The time evolution of WSS and vorticity in the RA model showed similar trends (Fig 12). A correlation is present between WSS and vorticity, quantified based on a Pearson correlation coefficient of 0.87.

**Fig 13:**
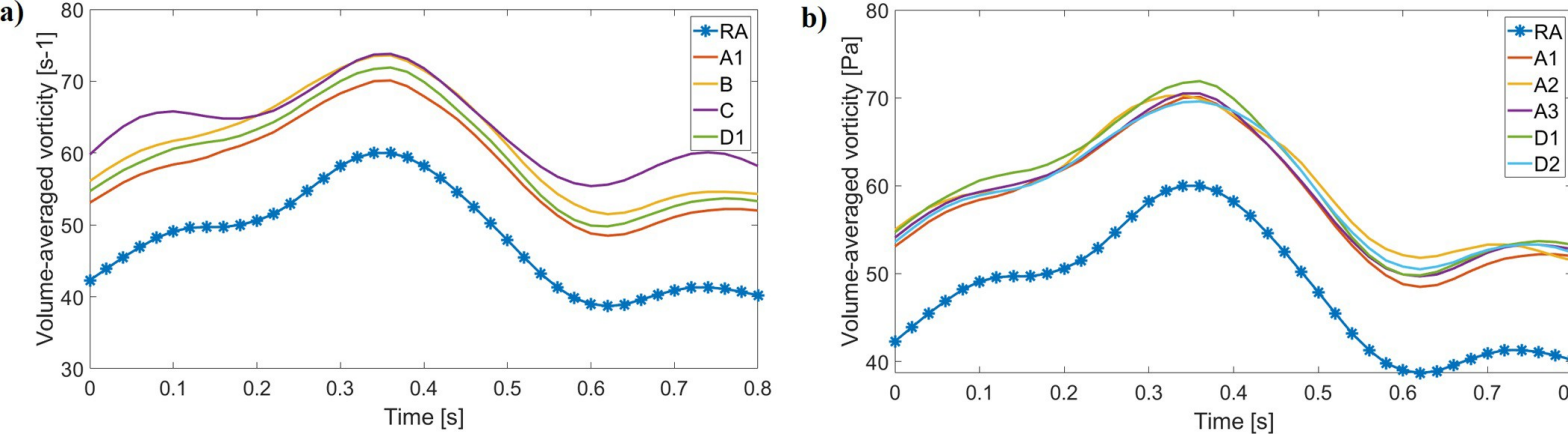
Volume-averaged vorticity profile through the cardiac cycle for the RA and all catheter models (generated with data from S12 File). (a) All designs are compared with the RA; (b) A and D tip placement changes do not greatly influence overall vorticity quantifications.

Time and volume-averaged vorticity increased in all catheter models in comparison with the RA model (Table 7 and Fig 13A). Catheter designs yielded similar vorticity values (54–57.20 s^-1^), with a 29.71% maximum increase from RA vorticity. Catheter C had the greatest average flow vorticity, as indicated by increased vorticity values during early systole and late diastole in comparison with other designs (Fig 13A). Changing the catheter tip placement or rotating it did not greatly impact the average RA vorticity for designs A and D (Fig 13B), yielding absolute average differences of 2.22% and 1.85%, respectively.

As observed in Table 7, time-averaged WSS on the RA wall was not markedly affected for catheters B and C. Catheters A1, A2 and A3 suffered time-averaged WSS percentage increases ranging between 6 and 9%, while catheters D1 and D2 had the greatest percentage increases (13–14%), in comparison with the value obtained for the RA model.

**Fig 14:**
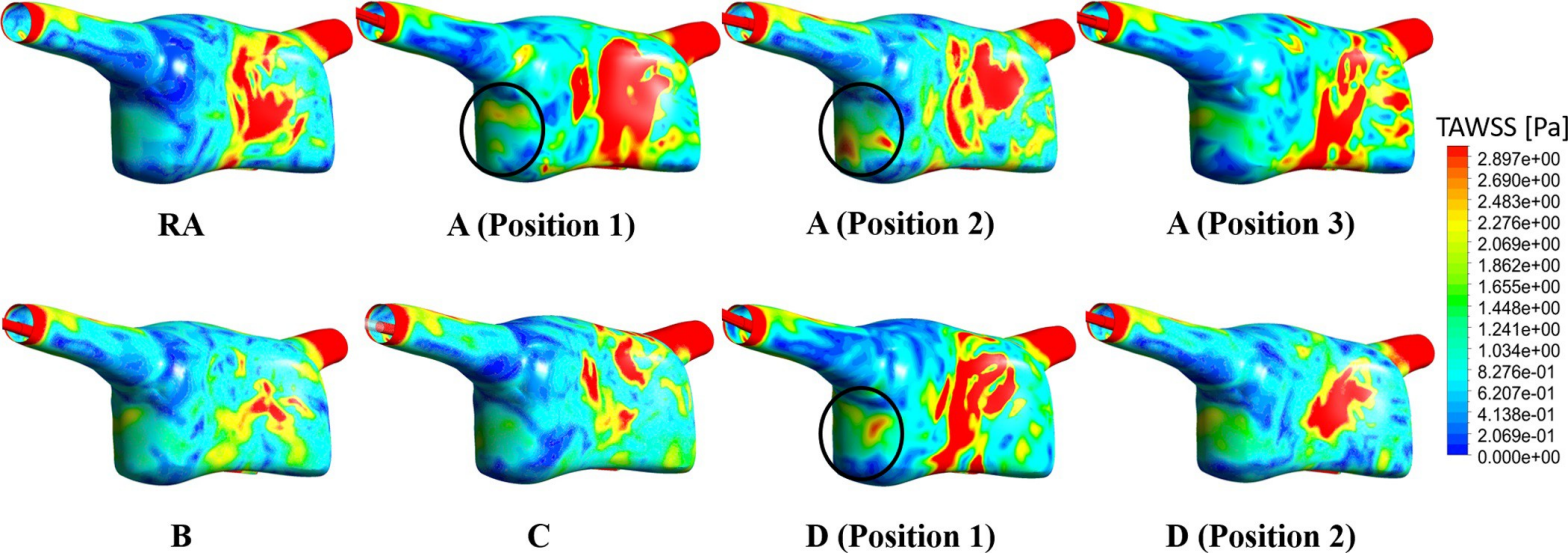
Time-averaged WSS [Pa] for the whole RA domain.

Time-averaged WSS magnitude distributions are presented on Fig 14. Areas with elevated WSS are located around the SVC and IVC inlets in all models, possibly due to local diameter reductions. Both sites of low and high magnitudes are present on the wall of the RA model, with high magnitude locations possibly corresponding to regions of elevated vorticity and helicity. The central region of the RA has sites of increased WSS magnitude for all models, whose surface distribution changes in the catheter models according to the blood flow jet pattern from each catheter tip. Moreover, designs A and D were associated with different sites of increased WSS, as observed below the SVC junction (circled on Fig 14).

#### Analysis of recirculation

**Fig 15:**
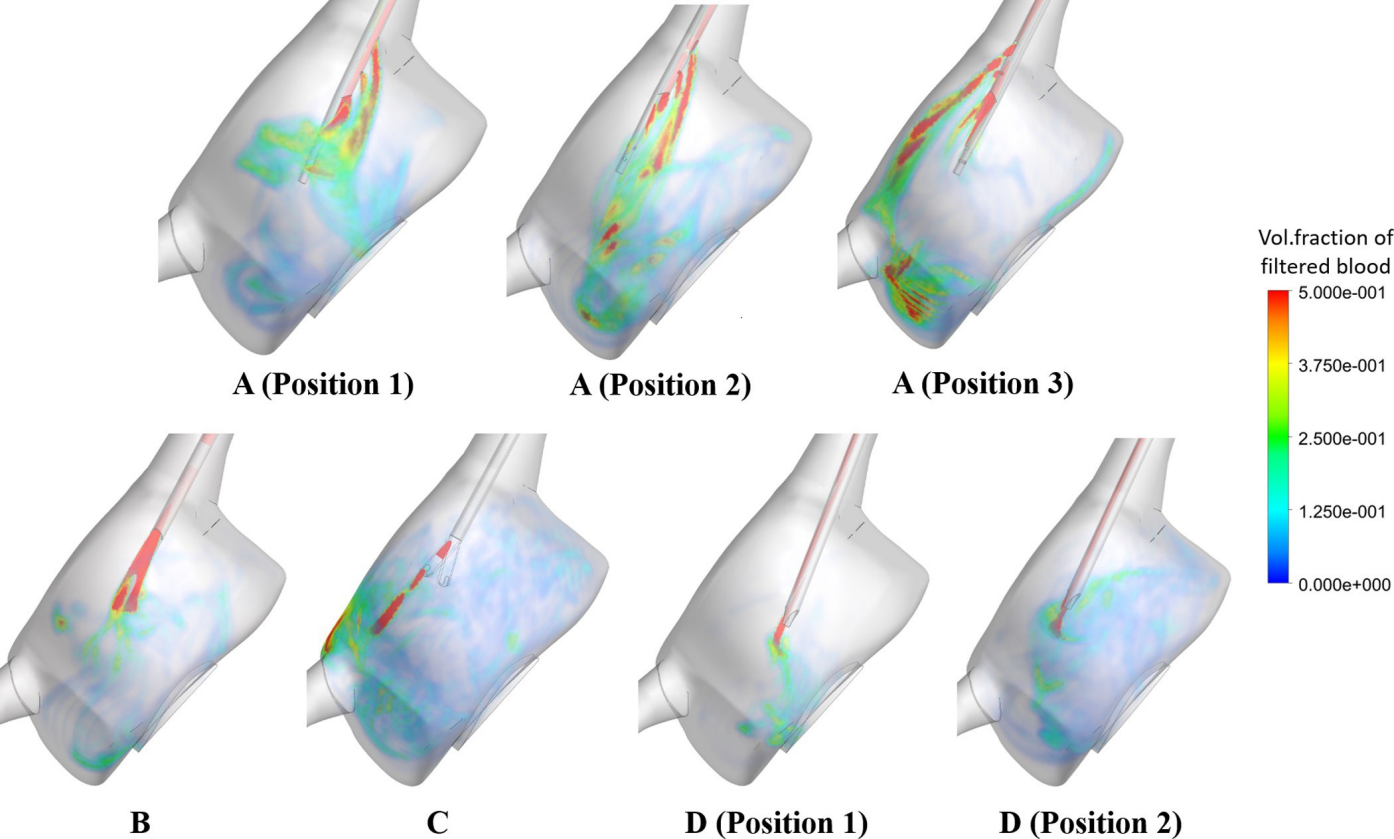
Time-averaged volume fraction of filtered blood (recirculation phase) within the RA for all catheter models.

All catheter models yielded flow recirculation (Table 7): while B was associated with the highest recirculation percentage (43.7%), A and C yielded recirculation > 5%. Fig 13 provides further information on the performance of these catheters: the side holes present in A allow for venous lumen flow to enter the RA with different trajectories, which seems to assist in a better mixing of this flow with the chamber flow and prevents it from entering the arterial lumen. The lack of side holes in B, however, seems to enhance the amount of flow returning through the catheter arterial lumen. Design D gave rise to the lowest percentages of recirculating fluid (< 0.30%), meeting the design requirements of less than 1% recirculation. Varying tip placement for design D did not greatly impact on recirculation percentages. On the other hand, Fig 15 shows that C and D (Position 2) are associated with the greatest mixing of venous flow within the RA, in comparison with the other designs.

Recirculation values were also altered with different tip placements of A; 30.79% and 5.15% decreases were observed for Positions 2 and 3 in comparison with the first position, respectively. Moreover, the mixing structures within the RA were altered, with Positions 2 and 3 giving rise to a greater concentration of venous flow at the base of the RA and near the IVC junction, respectively.

#### Analysis of shear stress

**Fig 16:**
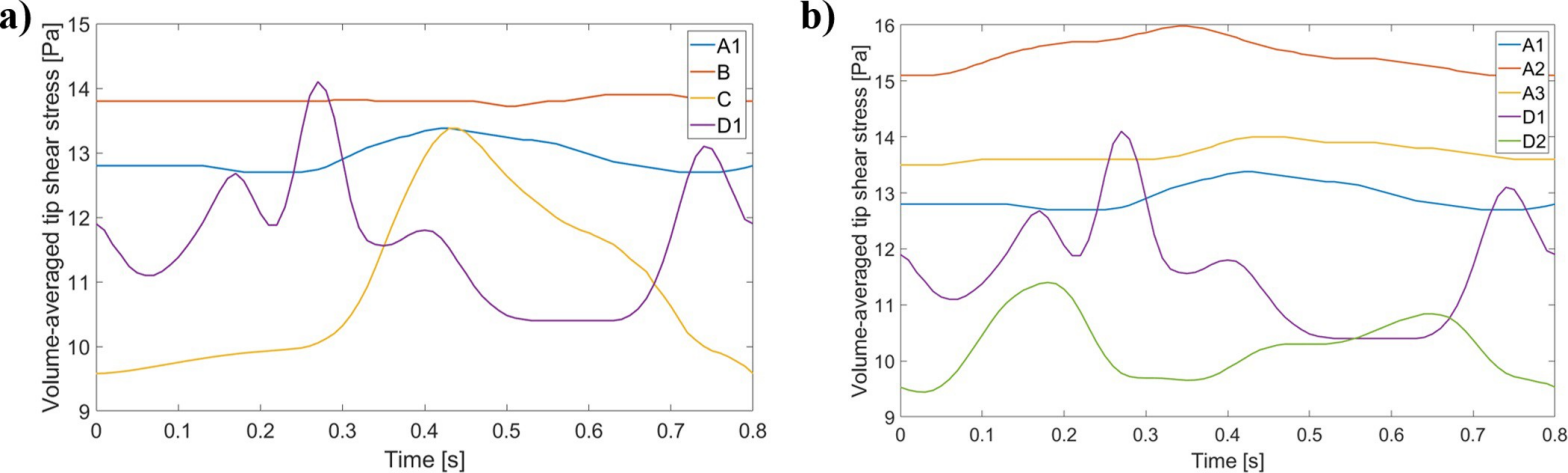
Volume-averaged shear stress profile through the cardiac cycle for all catheter venous lumen tips (generated with data from S13 File). (a) All designs are present; (b) A and D tip placement changes impact on tip shear stress.

**Fig 17:**
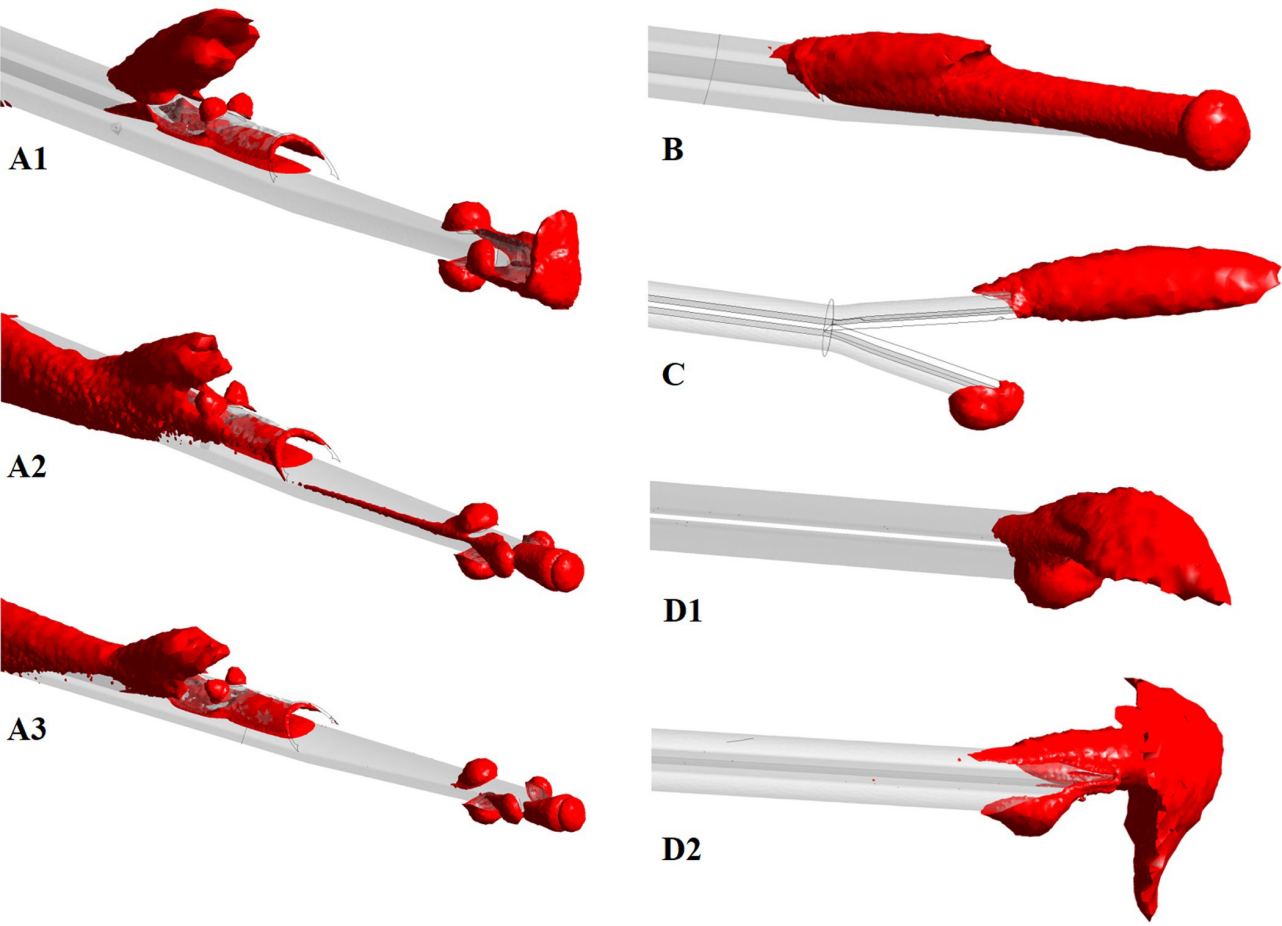
Isosurface regions where blood shear stress is 10 Pa for all models at the beginning of systole t = 0.25 s.

C and D catheter models were characterized by the lowest volume time-averaged shear stress at the tip, while the A and B designs led to the highest ones (Table 7). C was associated with a small percentage of volume of τ > 10 Pa (15.70%), while the other designs yielded values in the same range (above 28%). Moreover, and as observed in Table 7 and Fig 16B, changing tip placement affected the predicted shear stress for A and D. A tip placement closer to the atrium wall (Position 2) increased the volume-averaged shear stress and percentage of volume of τ > 10 Pa for design A by 20.15% and 18.02%, respectively, but decreased these quantities for design D by 12.07% and 0.70%, respectively. Rotating catheter A (Position 3) greatly improved these outcomes, but there was still an increase of 6.20% in the volume-averaged shear stress and of 1.06% on the percentage of volume of τ > 10 Pa in comparison with the first placement. The shear stress profile from Fig 16B captures similar temporal trends, and interestingly, for design D, the first position is associated with unsteady shear stress through time (also observed on Fig 16A, while the second one yields smoother shear stress changes. Fig 16A also shows that, while A and B designs yield relatively constant temporal shear stress, for C this stress increased near end-systole and decreased through diastole. Shear stress equal to 10 Pa were localized to both inlet and outlet boundaries, as well as side-holes (Fig 17). The venous lumen exit, however, had a greater proportion of shear stress equal to 10 Pa for all catheters.

## Discussion

### Main study findings

This is the first study to develop and validate a CFD model of the RA to assess catheter design performance. Key haemodynamics have been quantified for four distinct catheter designs. The obtained results suggest the following findings:

- The haemodynamic predictions for the RA model are consistent with *in vivo* data available in literature [42, 45, 48] and with clinical guidelines for right heart assessment [44], therefore, verifying our computational model;
- Catheter insertion induces increased vorticity and alterations in time-averaged WSS in RA haemodynamics;
- Recirculation is present in all catheter designs, with only the symmetric design D complying with required specifications (< 1%);
- The presence of side holes decreases the amount of recirculating flow in step designs, as given by lower recirculation percentages (6.45–9.32%) in design A when compared to design B (43.7%);
- Catheters working in reverse mode (step designs) are associated with reduced performance, assessed through greater recirculation percentages and average shear stress values;
- Elevated tip shear stress (10.20–15.50 Pa) is present in catheter designs, which can induce platelet activation and aggregation and subsequently thrombosis [51];
- Different catheter tip placements impact on performance, as given by altered recirculation percentages, tip shear stress values and percentage of tip volume with τ > 10 Pa;
- The catheter design with best performance is the symmetric one, associated with low recirculation and shear stress values.

### Computational model validation

Both the function and geometry of the RA are heterogeneous across patients [42, 45]. This variability has not been evaluated in our study, instead we assumed our RA model to provide a representative model of the RA (with/without dialysis catheters). The advantage of this approach is that it enables a direct comparison of the range of catheters evaluated (including positioning).

Similarly to previous heart modelling studies, the outlet was not extended as the location of the valve was assumed as the outflow boundary [19, 52], as extending the outlet boundary beyond the location of the valve would give rise to incorrect computational predictions as a valve is found at that position (e.g. alteration of pressure gradients near the valve). Moreover, the SVC and IVC inlets are placed 4 and 2 diameters away from the main chamber, respectively. To assess the independence of computational predictions from the domain size, additional simulations were solved for the RA model, with this having the IVC extended and the IVC inlet placed at 4 diameters away from the main chamber. Volume-averaged velocity and vorticity quantities, as well as area-averaged WSS, were evaluated and average relative errors between predictions with and without IVC extension were 4.77%, 4.52% and 2.09% for velocity, vorticity and WSS, respectively. These relative errors are deemed acceptable and justify the choice of our RA domain for computational simulations.

Although blood flow through the RA was assumed laminar, it is known that, for some blood vessels (cranial artery bifurcation, for example), turbulence occurs at Reynolds numbers as low as 400 [53]. However, maximum calculated Reynolds numbers were lower than this value, and so the laminar assumption was considered valid. An advantage of using a RA CFD model to evaluate catheter performance is the possibility to assess the interaction between flow exiting the venous lumen of the catheter and the atrial wall, which is not feasible with simplified models [20]. However, *in silico* and *in vitro* right atrial flow literature is limited [42, 45], which does not enable extensive model validation. *In vivo* data for the RA, on the other hand, is based on small data sets with inter-individual variability [42]. Nonetheless, the comparison of our RA model haemodynamic predictions with *in vivo* measurements was considered fundamental and *in vitro/in silico* results were second choices for validation, due to the fact that any obtained data is subject to individual experimental bias or computational assumptions.

The choice of catheter inlet and outlet flow rates and pressures was based on literature values: clinically realistic flow rates range between 200 and 400 ml/min [5]. Further insight on catheter performance under varying flow rates can be considered future work. Moreover, on the venous side, blood flow through the catheter is driven by the positive pressure generated by the blood pump, while on the arterial side the driving force is the negative pressure generated by the same pump. This means that pressure values typically range between -250 and +250 mmHg on arterial and venous sides, respectively, justifying our boundary condition choices for catheter models [5]. On the other hand, the catheter structure was assumed rigid, with our model not accounting for catheter tip movement during the cardiac cycle, which is significant for split tips working in reverse mode [11]. A fluid-structure interaction approach for catheter deformation inside the RA haemodynamic environment needs to be employed; however, this type of approach is challenging and its application towards the study of catheter performance remains a current open problem.

### Vorticity and WSS in the RA are altered with catheter insertion

Temporal WSS magnitudes were statistically correlated with vorticity in the RA model, which shows that tracking the temporal behaviour of vortex structures may provide complimentary information on the WSS [43]. Similar to previous studies, we can hypothesize that the influence of rinsing motion of increasing vortex during the systolic phase is associated with increasing WSS, thereby avoiding thrombus formation within the RA [43]. Predicted time-averaged WSS values, however, were higher than those previously obtained by a 4D cardiac MRI study [48]. However, the accuracy of MRI measurements is narrowed by low temporal and spatial resolutions of the order of 40 ms and 2 mm^3^, respectively [54]. This causes an averaging of the *in vivo* measured velocity field, yielding spurious errors in the calculated velocity gradients at the wall-blood interface [54, 55]. Therefore, MRI-derived WSS magnitude values are usually underestimated [54], which makes computational modelling advantageous in this matter [55].

Increased vorticity, either time-averaged or through the cardiac cycle, was found in all catheter models with respect to the RA model. No previous studies on catheter design performance have analysed this aspect; however, efforts have been made to understand the connection between vorticity and cardiac function and efficiency [45]. As mentioned, the presence of vortices in the healthy RA seems to optimise blood flow and cardiac efficiency within the right heart [42, 45]. However, previous work has shown: increased right atrial vorticity in patients after repair of Tetralogy of Fallot, with these possessing additional diastolic vortices that impacted on right ventricular flow [45]; and altered right atrial vorticity in patients with right ventricular diastolic dysfunction [56]. Fenster *et al*. (2015) suggested that, although premature, quantifying vorticity could be useful to: 1) correlate to right heart pathologies, and 2) serve as a non-invasive biomarker for the assessment of both haemodynamic and bioenergetics response to therapy [56]. Based on our results and the outcomes of previous studies, we speculate that catheter insertion in the RA may alter normal blood flow within this chamber [48]. However, objective clinical outcomes derived from this speculative hypothesis would have to be further studied.

Previous clinical [57] and computational [58, 59] studies have linked abnormal flow with high WSS, as well as different WSS distributions, in the ascending aorta. Abnormally high WSS has, in fact, been assumed as a trigger for aortic dilation in congenital diseases by changing the wall tissue mechanical properties [60–62]. Our results show a change in time-averaged WSS, as well as magnitude distributions in the atrial wall surface, with catheter insertion in the RA. Indeed, the symmetric tip (design D) was associated with the highest increase. Moreover, step and symmetric tips (designs A and D) gave rise to the creation of different high WSS sites. Given the previous findings mentioned above, we can hypothesize that these WSS alterations could possibly be associated with right atrial enlargement onset and progression at specific sites.

### Catheter performance

Predicted time-averaged recirculation values for all models are within the same range as those obtained by previous studies [11–13]. Similarly to a previous study, the symmetric design (D) yielded negligible (< 0.5%) recirculation [13], and the lowest of all models, which is associated with a better separation of filtered and unfiltered blood. Moreover, designs working in reverse mode (step tips) gave rise to higher recirculation percentages, in comparison with symmetric and split designs. In fact, the literature shows the presence of up to 86% of recirculation for catheters working in reverse configuration [11, 63], which can validate our highest recirculation value (43.7%) for step design B. Moreover, and similarly to previous work [11, 13], the presence of side holes in step tip design (A and B) impacted in recirculation rates by diminishing time-averaged recirculating flow. We hypothesize that the presence of side holes in a step tip may improve performance by allowing flow deflection from the distal tip, as suggested elsewhere [13].

Shear stress characteristics were also evaluated to study the tendency of each catheter design to cause shear-induced platelet activation and aggregation. Platelet activation has been shown to induce device thrombogenicity and they experience shear-induced activation at a larger rate than what is required for haemolysis of red blood cells [64]. Our predicted shear stresses were in the same order of magnitude as presented elsewhere, although percentages of tip volume above 10 Pa were much lower [14]. This could, however, be due to the variability in tip volume definition. Nonetheless, platelet activation has been observed with 0.1 ≤ τ ≤ 20 Pa [51]. Here, we used a middle value as a threshold (10 Pa), not being overly conservative, as it is a relative measure which is being used ultimately to compare catheters.

All catheter models yielded shear stress above 10 Pa, and such stress was mainly observed at the venous tip and side holes in the step design. The elevated shear stress location was similar to that observed in a previous *in vivo* study, which showed the formation of a fibrin plaque on catheter surface around a venous side hole [65]. This suggests that all designs have a potential for shear-induced platelet activation and subsequently thrombosis.

Catheters working in reverse mode (step tip designs) yielded the highest recirculation and time-averaged shear stress values. According to this, the highest potential for shear-induced platelet activation and worse recirculation outcomes were observed for step designs in reverse mode, implying that, in a clinical scenario, the use of standard mode should be targeted. The symmetric tip, however, was shown to have the best performance, as given by the lowest recirculation and shear stress values.

Different tip placements also yielded different recirculation percentages, with a tip placement closer to the RA wall giving rise to greater recirculating flow. This is the first computational study which considers how different catheter tip positions impact on the respective performance, as well as how they affect flow within the RA. However, the optimal positioning of an haemodialysis catheter is a continuous subject of debate, with elevated clinical variability [66] and changing medical guidelines [27]. Previous studies noted that this positioning is crucial to prevent/diminish recirculation [67] and it is known that such percentage impacts on the efficiency of the haemodialysis treatment [68]. Per our results, a tip placement at the mid-level of the RA with its arterial lumen facing the mediastinum yields lower (but still significant) recirculation percentages, which is in agreement with the latest medical guidelines [69]. Given this, care should be taken with catheter tip placement and orientation within the RA.

### Limitations

There are several limitations in this study, with most arising from the rigid walls and geometry of the RA reconstructed from dimensions in literature. Whilst such reconstruction lacks physiological accuracy, it presents a superior platform for quantitative comparisons of catheter performance compared to previously cylindrical models. Moreover, the RA model was used to mimic a healthy case, which may not be accurate for dialysis patients, especially concerning measures as central pressure [70]. Nonetheless, the use of such a model for the study of multiple catheter designs enables direct and objective comparison of their performance, including the evaluation of blood mixing (between blood filtered through dialysis and blood which was not filtered), not feasible through a cylinder model.

The absence of atrial contraction and TV opening/closing during the cardiac cycle was a major limitation, since the movement of the RA walls affects the blood flow patterns and mixing within the chamber, affecting catheter implementation. By employing rigid RA walls, and having into account the mass conservation balance, all flow entering this chamber must be equal to the flow exiting the TV, which is not physiologically accurate. Moreover, fixed-wall simulations oversimplify inflow-outflow boundary conditions and ignore atrial-ventricular interactions; they have been shown to yield different flow fields and stasis maps [71] and overestimate instantaneous quantities (e.g. flow velocity; WSS) when compared to moving wall simulations [72]; however, time-averaged quantities have been shown not to greatly vary [73]. The use of a fixed RA model may explain the overestimation of the velocity of blood flow through the IVC and SVC, their flow rate and the elevated WSS magnitudes observed in those vessels.

Whilst including RA wall motion may improve flow/pressure predications [74], very few studies provide insight on RA wall movement [75, 76] and experimental data on the mechanical properties of the RA wall is currently limited: previous studies mention that *in vivo* properties for the heart walls can be up to four orders of magnitude different from *ex vivo* properties [77]. Incorporating a non-rigid wall would, therefore, introduce a range of variables for which data is currently lacking. Given that RA models are still an emerging method, and despite these limitations, fixed-wall simulations can still be useful to assess the essential characteristics of blood flow within the RA.

The SVC blood flow rate waveform and maximum value were overestimated in comparison with the literature. However, there is elevated variability in patient data for blood flow rate through the SVC and IVC: for example, the difference between average and the maximum standard deviation values for the IVC in literature is 33% [49]. Moreover, since other hemodynamic features in the RA from our study are consistent with the literature, we accept this as a limitation of the study which does not greatly impact on the remaining computational predictions concerning the evaluation of catheter performance, especially since all catheters have been compared using the same model.

The function of the TV was not modelled. This yielded a different flow rate waveform in comparison with those from the literature [50], potentially giving rise to flow patterns and mixing within the RA which differ from a model with valve opening/closing. Moreover, the Neumann assumption may not have been satisfied during the diastolic period, when the TV is open, due to the location of the outlet and valve function not being included; however, it is when the valve is closed during the systole. Including TV function in the model would most likely lead to a more accurate flow rate waveform through the cardiac cycle, as well as increase the pressure drop due to valve closure (which could increase the range of variation of pressure values through one cardiac cycle). Moreover, valve closure would allow the RA to act as a better “reservoir” during systole, leading to a greater flow volume within the RA during this period (when the TV is closed). This could enhance the presence of vortices within the RA (increasing vorticity predictions during systole) and possibly give rise to retrograde flow in the cava veins. Nonetheless, our study focuses on the use of a RA model to evaluate catheter designs and incorporating a fluid-structure interaction valve model would fall outside its scope. In addition, our model enabled good approximations of flow velocity and flow patterns within the RA, in comparison with the literature. Our current RA model has also enabled the study of catheter performance including evaluation of mixing of blood (at the level of the TV), which had, and had not, been filtered via haemodialysis.

## Conclusion

In this study we present a model which provides realistic predictions of haemodynamics in the right atrium, subsequently aiding assessment of haemodialysis catheter performance. Our model shows that the symmetric tip design is associated with the best haemodynamic results, given by its low recirculation and shear stress values, while the step tip designs working in reverse mode gave rise to the worst haemodynamic outcomes. Moreover, the presence of side holes at the tip helped diminish recirculating flow, suggesting that, in the design process of a step tip, this feature should be looked into to improve its performance. In addition, catheter performance was affected by different tip placements, showing that correct positioning should be accounted for when placing the device in the RA.

## Supporting information

S1 File STL file representing the geometry of catheter A. (STL)

S2 File STL file representing the geometry of catheter B. (STL)

S3 File STL file representing the geometry of catheter C. (STL)

S4 File STL file representing the geometry of catheter D. (STL)

S5 File Probe location for the mesh independence study. (DOCX)

S6 File Values and calculations for the mesh independence study. (DOCX)

S7 File Comparison of Newtonian and non-Newtonian Bird-Carreau models. (DOCX)

S8 File C code that generates the inlet boundary condition pressure waveform. (C)

S9 File Velocity and pressure values used for temporal convergence study. (XLSX)

S10 File Velocity, area, and respective flow rate values used to generate flow rate temporal profiles at SVC, IVC and TV boundaries. (XLSX)

S11 File Spatially averaged WSS and volume-averaged vorticity values for the RA model. (XLSX)

S12 File Volume-averaged vorticity values for all models. (XLSX)

S13 File Volume-averaged shear stress values for all catheter models. (XLSX)

## References

[1] Smith RN, Nolan JP. Central venous catheters. BMJ. 2013;347:f6570. 10.1136/bmj.f6570 24217269

[2] Malas MB, Canner JK, Hicks CW, Arhuidese IJ, Zarkowsky DS, Qazi U, et al. Trends in incident hemodialysis access and mortality. JAMA Surg. 2015;150(5):441–8. 10.1001/jamasurg.2014.3484 25738981

[3] Collins AJ, Foley RN, Chavers B, Gilbertson D, Herzog C, Johansen K, et al. ’United States Renal Data System 2011 Annual Data Report: Atlas of chronic kidney disease & end-stage renal disease in the United States. Am J Kidney Dis. 2012;59(1 Suppl 1):A7, e1-420. 10.1053/j.ajkd.2011.11.015 22177944

[4] Knuttinen MG, Bobra S, Hardman J, Gaba RC, Bui JT, Owens CA. A review of evolving dialysis catheter technologies. Semin Intervent Radiol. 2009;26(2):106–14. 10.1055/s-0029-1222453 21326500 PMC3036434

[5] Depner TA. Catheter performance. Semin Dial. 2001;14(6):425–31. 10.1046/j.1525-139x.2001.00106.x 11851927

[6] Dhingra RK, Young EW, Hulbert-Shearon TE, Leavey SF, Port FK. Type of vascular access and mortality in U.S. hemodialysis patients. Kidney Int. 2001;60(4):1443–51. 10.1046/j.1523-1755.2001.00947.x 11576358

[7] Lok CE, Foley R. Vascular access morbidity and mortality: trends of the last decade. Clin J Am Soc Nephrol. 2013;8(7):1213–9. 10.2215/CJN.01690213 23824198

[8] Kakkos SK, Haddad GK, Haddad RK, Scully MM. Effectiveness of a new tunneled catheter in preventing catheter malfunction: a comparative study. J Vasc Interv Radiol. 2008;19(7):1018–26. 10.1016/j.jvir.2008.03.006 18589315

[9] Tal MG. Comparison of recirculation percentage of the palindrome catheter and standard hemodialysis catheters in a swine model. J Vasc Interv Radiol. 2005;16(9):1237–40. 10.1097/01.RVI.0000171700.45582.9E 16151065

[10] Foust J. Blood flow simulation past a catheter positioned in the SVC-IVC-RA junction: steady and unsteady flow considerations: Lehigh University; 2004.

[11] Vesely TM, Ravenscroft A. Hemodialysis catheter tip design: observations on fluid flow and recirculation. J Vasc Access. 2016;17(1):29–39. 10.5301/jva.5000463 26349860

[12] Clark TW, Van Canneyt K, Verdonck P. Computational flow dynamics and preclinical assessment of a novel hemodialysis catheter. Semin Dial. 2012;25(5):574–81. 10.1111/j.1525-139X.2012.01052.x 22353667

[13] Clark TWI, Isu G, Gallo D, Verdonck P, Morbiducci U. Comparison of Symmetric Hemodialysis Catheters Using Computational Fluid Dynamics. J Vasc Interv Radiol. 2015;26(2):252–9. 10.1016/j.jvir.2014.11.004 25645414

[14] Mareels G, Kaminsky R, Eloot S, Verdonck PR. Particle image velocimetry-validated, computational fluid dynamics-based design to reduce shear stress and residence time in central venous hemodialysis catheters. ASAIO J. 2007;53(4):438–46. 10.1097/MAT.0b013e3180683b7c 17667228

[15] Einstein DR, Del Pin F, Jiao X, Kuprat AP, Carson JP, Kunzelman KS, et al. Fluid-Structure Interactions of the Mitral Valve and Left Heart: Comprehensive Strategies, Past, Present and Future. Int J Numer Methods Eng. 2010;26(3–4):348–80. 10.1002/cnm.1280 20454531 PMC2864615

[16] Gao H, Feng L, Qi N, Berry C, Griffith BE, Luo X. A coupled mitral valve-left ventricle model with fluid-structure interaction. Med Eng Phys. 2017;47:128–36. 10.1016/j.medengphy.2017.06.042 28751011 PMC6779302

[17] Adham Esfahani S, Hassani K, Espino DM. Fluid-structure interaction assessment of blood flow hemodynamics and leaflet stress during mitral regurgitation. Comput Methods Biomech Biomed Engin. 2019;22(3):288–303. 10.1080/10255842.2018.1552683 30596526

[18] Mao W, Caballero A, McKay R, Primiano C, Sun W. Fully-coupled fluid-structure interaction simulation of the aortic and mitral valves in a realistic 3D left ventricle model. PLoS One. 2017;12(9):e0184729. 10.1371/journal.pone.0184729 28886196 PMC5590990

[19] Rigatelli G, Zuin M, Fong A. Computational Flow Dynamic Analysis of Right and Left Atria in Patent Foramen Ovale: Potential Links with Atrial Fibrillation. J Atr Fibrillation. 2018;10(5):1852. 10.4022/jafib.1852 29988264 PMC6006970

[20] Mareels G, De Wachter DS, Verdonck PR. Computational fluid dynamics-analysis of the Niagara hemodialysis catheter in a right heart model. Artif Organs. 2004;28(7):639–48. 10.1111/j.1525-1594.2004.07371.x 15209857

[21] Patil S, Jadhav S, Shetty N, Kharge J, Puttegowda B, Ramalingam R, et al. Assessment of inferior vena cava diameter by echocardiography in normal Indian population: A prospective observational study. Indian Heart J. 2016;68 Suppl 3:S26–S30. 10.1016/j.ihj.2016.06.009 28038721 PMC5198879

[22] Sonavane SK, Milner DM, Singh SP, Abdel Aal AK, Shahir KS, Chaturvedi A. Comprehensive Imaging Review of the Superior Vena Cava. Radiographics. 2015;35(7):1873–92. 10.1148/rg.2015150056 26452112

[23] Ton-Nu TT, Levine RA, Handschumacher MD, Dorer DJ, Yosefy C, Fan D, et al. Geometric determinants of functional tricuspid regurgitation: insights from 3-dimensional echocardiography. Circulation. 2006;114(2):143–9. 10.1161/CIRCULATIONAHA.106.611889 16818811

[24] Huttin O, Voilliot D, Mandry D, Venner C, Juilliere Y, Selton-Suty C. All you need to know about the tricuspid valve: Tricuspid valve imaging and tricuspid regurgitation analysis. Arch Cardiovasc Dis. 2016;109(1):67–80. 10.1016/j.acvd.2015.08.007 26711544

[25] Maceira AM, Cosin-Sales J, Roughton M, Prasad SK, Pennell DJ. Reference right atrial dimensions and volume estimation by steady state free precession cardiovascular magnetic resonance. J Cardiovasc Magn Reson. 2013;15:29. 10.1186/1532-429X-15-29 23566426 PMC3627628

[26] Sievers B, Addo M, Breuckmann F, Barkhausen J, Erbel R. Reference right atrial function determined by steady-state free precession cardiovascular magnetic resonance. J Cardiovasc Magn Reson. 2007;9(5):807–14. 10.1080/10976640701545552 17891619

[27] Tawk S, Barakat E, Hammer F. A Proposed Simple and Accurate Technique for Optimal Long-Term Hemodialysis Catheter Tip Placement. J Belg Soc Radiol. 2018;102(1):21. 10.5334/jbsr.1474 30039035 PMC6032382

[28] Shewchuk JR, editor What is a Good Linear Element? Interpolation, Conditioning, and Quality Measures. Eleventh International Meshing Roundtable; 2002.

[29] Workbench A. ANSYS Meshing User’s Guide 16.0. Ansys Inc, USA; 2015.

[30] Owen DG, Schenkel T, Shepherd DET, Espino DM. Assessment of surface roughness and blood rheology on local coronary haemodynamics: a multi-scale computational fluid dynamics study. J R Soc Interface. 2020;17(169):20200327. 10.1098/rsif.2020.0327 32781935 PMC7482556

[31] Owen DG, de Oliveira DC, Qian S, Green NC, Shepherd DET, Espino DM. Impact of side-hole geometry on the performance of hemodialysis catheter tips: A computational fluid dynamics assessment. PLoS One. 2020;15(8):e0236946. 10.1371/journal.pone.0236946 32764790 PMC7413473

[32] Carty G, Chatpun S, Espino DM. Modeling Blood Flow Through Intracranial Aneurysms: A Comparison of Newtonian and Non-Newtonian Viscosity. J Med Biol Eng. 2016;36(3):396–409.

[33] Cho YI, Kensey KR. Effects of the non-Newtonian viscosity of blood on flows in a diseased arterial vessel. Part 1: Steady flows. Biorheology. 1991;28(3–4):241–62. 10.3233/bir-1991-283-415 1932716

[34] Johnston BM, Johnston PR, Corney S, Kilpatrick D. Non-Newtonian blood flow in human right coronary arteries: steady state simulations. J Biomech. 2004;37(5):709–20. 10.1016/j.jbiomech.2003.09.016 15047000

[35] Valen-Sendstad K, Mardal KA, Mortensen M, Reif BA, Langtangen HP. Direct numerical simulation of transitional flow in a patient-specific intracranial aneurysm. J Biomech. 2011;44(16):2826–32. 10.1016/j.jbiomech.2011.08.015 21924724

[36] Cohen ML, Cohen BS, Kronzon I, Lighty GW, Winer HE. Superior vena caval blood flow velocities in adults: a Doppler echocardiographic study. J Appl Physiol (1985). 1986;61(1):215–9. 10.1152/jappl.1986.61.1.215 3733606

[37] Parker K, Thiriet M. Physiology and pathology of the cardiovascular system: a physical perspective. In: Formaggia L, Quarteroni A, Veneziani A, editors. Cardiovascular Mathematics Modeling and simulation of the circulatory system. Milano: Springer; 2009. p. 1–47.

[38] ANSYS I. ANSYS Fluent Tutorial Guide (Release 18.1). Release 18.0 ed. 275 Technology Drive Canonsburg PA 15317: ANSYS, Inc.; 2017.

[39] Espino DM, Shepherd DET, Hukins DWL. Transient large strain contact modelling: A comparison of contact techniques for simultaneous fluid-structure interaction. Eur J Mech B-Fluid. 2015;51:54–60.

[40] ANSYS I. ANSYS Fluent Theory Guide (Release 15.0). Release 15.0 ed. 275 Technology Drive Canonsburg PA 15317: ANSYS, Inc.; 2013.

[41] Wang JS, Li YS, Chen JC, Chen YW. Effects of exercise training and deconditioning on platelet aggregation induced by alternating shear stress in men. Arterioscler Thromb Vasc Biol. 2005;25(2):454–60. 10.1161/01.ATV.0000151987.04607.24 15569820

[42] Parikh JD, Kakarla J, Keavney B, O’Sullivan JJ, Ford GA, Blamire AM, et al. 4D flow MRI assessment of right atrial flow patterns in the normal heart—influence of caval vein arrangement and implications for the patent foramen ovale. PLoS One. 2017;12(3):e0173046. 10.1371/journal.pone.0173046 28282389 PMC5345792

[43] Gulan U, Saguner A, Akdis D, Gotschy A, Manka R, Brunckhorst C, et al. Investigation of Atrial Vortices Using a Novel Right Heart Model and Possible Implications for Atrial Thrombus Formation. Sci Rep-Uk. 2017;7. 10.1038/s41598-017-17117-3 29196688 PMC5711865

[44] Rudski LG, Lai WW, Afilalo J, Hua L, Handschumacher MD, Chandrasekaran K, et al. Guidelines for the echocardiographic assessment of the right heart in adults: a report from the American Society of Echocardiography endorsed by the European Association of Echocardiography, a registered branch of the European Society of Cardiology, and the Canadian Society of Echocardiography. J Am Soc Echocardiogr. 2010;23(7):685–713; quiz 86–8. 10.1016/j.echo.2010.05.010 20620859

[45] Hirtler D, Garcia J, Barker AJ, Geiger J. Assessment of intracardiac flow and vorticity in the right heart of patients after repair of tetralogy of Fallot by flow-sensitive 4D MRI. Eur Radiol. 2016;26(10):3598–607. 10.1007/s00330-015-4186-1 26747260 PMC4938791

[46] Elbaz MS, van der Geest RJ, Calkoen EE, de Roos A, Lelieveldt BP, Roest AA, et al. Assessment of viscous energy loss and the association with three-dimensional vortex ring formation in left ventricular inflow: In vivo evaluation using four-dimensional flow MRI. Magn Reson Med. 2017;77(2):794–805. 10.1002/mrm.26129 26924448 PMC5297883

[47] Kilner PJ, Yang GZ, Wilkes AJ, Mohiaddin RH, Firmin DN, Yacoub MH. Asymmetric redirection of flow through the heart. Nature. 2000;404(6779):759–61. 10.1038/35008075 10783888

[48] Francois CJ, Srinivasan S, Schiebler ML, Reeder SB, Niespodzany E, Landgraf BR, et al. 4D cardiovascular magnetic resonance velocity mapping of alterations of right heart flow patterns and main pulmonary artery hemodynamics in tetralogy of Fallot. J Cardiovasc Magn Reson. 2012;14:16. 10.1186/1532-429X-14-16 22313680 PMC3305663

[49] Wehrum T, Lodemann T, Hagenlocher P, Stuplich J, Ngo BTT, Grundmann S, et al. Age-related changes of right atrial morphology and inflow pattern assessed using 4D flow cardiovascular magnetic resonance: results of a population-based study. J Cardiovasc Magn Reson. 2018;20(1):38. 10.1186/s12968-018-0456-9 29898733 PMC6001162

[50] Kroft LJ, Simons P, van Laar JM, de Roos A. Patients with pulmonary fibrosis: cardiac function assessed with MR imaging. Radiology. 2000;216(2):464–71. 10.1148/radiology.216.2.r00jl06464 10924571

[51] Kroll MH, Hellums JD, McIntire LV, Schafer AI, Moake JL. Platelets and shear stress. Blood. 1996;88(5):1525–41. 8781407

[52] Vedula V, George R, Younes L, Mittal R. Hemodynamics in the Left Atrium and Its Effect on Ventricular Flow Patterns. J Biomech Eng. 2015;137(11):111003. 10.1115/1.4031487 26329022

[53] Roach MR, Scott S, Ferguson GG. The hemodynamic importance of the geometry of bifurcations in the circle of Willis (glass model studies). Stroke. 1972;3(3):255–67. 10.1161/01.str.3.3.255 5034974

[54] Rinaudo A, Pasta S. Regional variation of wall shear stress in ascending thoracic aortic aneurysms. Proc Inst Mech Eng H. 2014;228(6):627–38. 10.1177/0954411914540877 24942163

[55] Papathanasopoulou P, Zhao S, Kohler U, Robertson MB, Long Q, Hoskins P, et al. MRI measurement of time-resolved wall shear stress vectors in a carotid bifurcation model, and comparison with CFD predictions. J Magn Reson Imaging. 2003;17(2):153–62. 10.1002/jmri.10243 12541221

[56] Fenster BE, Browning J, Schroeder JD, Schafer M, Podgorski CA, Smyser J, et al. Vorticity is a marker of right ventricular diastolic dysfunction. Am J Physiol-Heart C. 2015;309(6):H1087–H93. 10.1152/ajpheart.00278.2015 26254331

[57] Mahadevia R, Barker AJ, Schnell S, Entezari P, Kansal P, Fedak PW, et al. Bicuspid aortic cusp fusion morphology alters aortic three-dimensional outflow patterns, wall shear stress, and expression of aortopathy. Circulation. 2014;129(6):673–82. 10.1161/CIRCULATIONAHA.113.003026 24345403 PMC3946057

[58] Oliveira D, Rosa SA, Tiago J, Ferreira RC, Agapito AF, Sequeira A. Bicuspid aortic valve aortopathies: An hemodynamics characterization in dilated aortas. Comput Methods Biomech Biomed Engin. 2019;22(8):815–26. 10.1080/10255842.2019.1597860 30957542

[59] Cao K, Sucosky P. Computational comparison of regional stress and deformation characteristics in tricuspid and bicuspid aortic valve leaflets. Int J Numer Method Biomed Eng. 2017;33(3). 10.1002/cnm.2798 27138991

[60] Tadros TM, Klein MD, Shapira OM. Ascending aortic dilatation associated with bicuspid aortic valve: pathophysiology, molecular biology, and clinical implications. Circulation. 2009;119(6):880–90. 10.1161/CIRCULATIONAHA.108.795401 19221231

[61] Bissell MM, Hess AT, Biasiolli L, Glaze SJ, Loudon M, Pitcher A, et al. Aortic dilation in bicuspid aortic valve disease: flow pattern is a major contributor and differs with valve fusion type. Circ Cardiovasc Imaging. 2013;6(4):499–507. 10.1161/CIRCIMAGING.113.000528 23771987 PMC3859916

[62] Barker AJ, Markl M, Burk J, Lorenz R, Bock J, Bauer S, et al. Bicuspid aortic valve is associated with altered wall shear stress in the ascending aorta. Circ Cardiovasc Imaging. 2012;5(4):457–66. 10.1161/CIRCIMAGING.112.973370 22730420

[63] Pannu N, Jhangri GS, Tonelli M. Optimizing dialysis delivery in tunneled dialysis catheters. Asaio Journal. 2006;52(2):157–62. 10.1097/01.mat.0000202081.13974.39 16557101

[64] Klaus S, Korfer S, Mottaghy K, Reul H, Glasmacher B. In vitro blood damage by high shear flow: human versus porcine blood. Int J Artif Organs. 2002;25(4):306–12. 10.1177/039139880202500409 12027141

[65] Lucas TC, Tessarolo F, Jakitsch V, Caola I, Brunori G, Nollo G, et al. Blood flow in hemodialysis catheters: a numerical simulation and microscopic analysis of in vivo-formed fibrin. Artif Organs. 2014;38(7):556–65. 10.1111/aor.12243 24341622

[66] Vesely TM. Central venous catheter tip position: a continuing controversy. J Vasc Interv Radiol. 2003;14(5):527–34. 10.1097/01.rvi.0000071097.76348.72 12761305

[67] Santoro D, Benedetto F, Mondello P, Pipito N, Barilla D, Spinelli F, et al. Vascular access for hemodialysis: current perspectives. Int J Nephrol Renovasc Dis. 2014;7:281–94. 10.2147/IJNRD.S46643 25045278 PMC4099194

[68] Twardowski ZJ, Seger RM. Measuring central venous structures in humans: implications for central-vein catheter dimensions. J Vasc Access. 2002;3(1):21–37. 10.1177/112972980200300105 17639457

[69] Gilmore J. KDOQI clinical practice guidelines and clinical practice recommendations—2006 updates. Nephrol Nurs J. 2006;33(5):487–8. 17044433

[70] Jean G, Chazot C, Vanel T, Charra B, Terrat JC, Calemard E, et al. Central venous catheters for haemodialysis: looking for optimal blood flow. Nephrol Dial Transplant. 1997;12(8):1689–91. 10.1093/ndt/12.8.1689 9269650

[71] García-Villalba M, Rossini L, Gonzalo A, Vigneault D, Martinez-Legazpi P, Flores O, et al. Demonstration of Patient-Specific Simulations To Assess Left Atrial Appendage Thrombogenesis Risk. 2020. 10.3389/fphys.2021.596596 PMC7953154 33716763

[72] Lopes D, Puga H, Teixeira JC, Teixeira SF. Influence of arterial mechanical properties on carotid blood flow: Comparison of CFD and FSI studies. Int J Mech Sci. 2019;160:209–18.

[73] Torii R, Wood NB, Hadjiloizou N, Dowsey AW, Wright AR, Hughes AD, et al. Fluid–structure interaction analysis of a patient‐specific right coronary artery with physiological velocity and pressure waveforms. Commun Numer Meth En. 2009;25(5):565–80.

[74] Formaggia L, Perktold K, Quarteroni A. Basic mathematical models and motivations. In: Formaggia L, Quarteroni A, Veneziani A, editors. Cardiovascular Mathematics: Modeling and simulation of the circulatory system: Springer; 2009. 10.1080/10255840903080802

[75] Vitarelli A, Mangieri E, Gaudio C, Tanzilli G, Miraldi F, Capotosto L. Right atrial function by speckle tracking echocardiography in atrial septal defect: Prediction of atrial fibrillation. Clin Cardiol. 2018;41(10):1341–7. 10.1002/clc.23051 30117180 PMC6489743

[76] Takata M, Harasawa Y, Beloucif S, Robotham JL. Coupled vs. uncoupled pericardial constraint: effects on cardiac chamber interactions. J Appl Physiol (1985). 1997;83(6):1799–813. 10.1152/jappl.1997.83.6.1799 9390949

[77] Rausch MK, Kuhl E. On the effect of prestrain and residual stress in thin biological membranes. J Mech Phys Solids. 2013;61(9):1955–69. 10.1016/j.jmps.2013.04.005 23976792 PMC3747014

