# Supplementary figures and images for: Computational fluid dynamics of the right atrium: Assessment of modelling criteria for the evaluation of dialysis catheters
Source: PLoS One. 2021 Feb 25;16(2):e0247438. doi: 10.1371/journal.pone.0247438 (PMC7906423; doi:10.1371/journal.pone.0247438)

**Location of probe within the RA (given by red mark):**


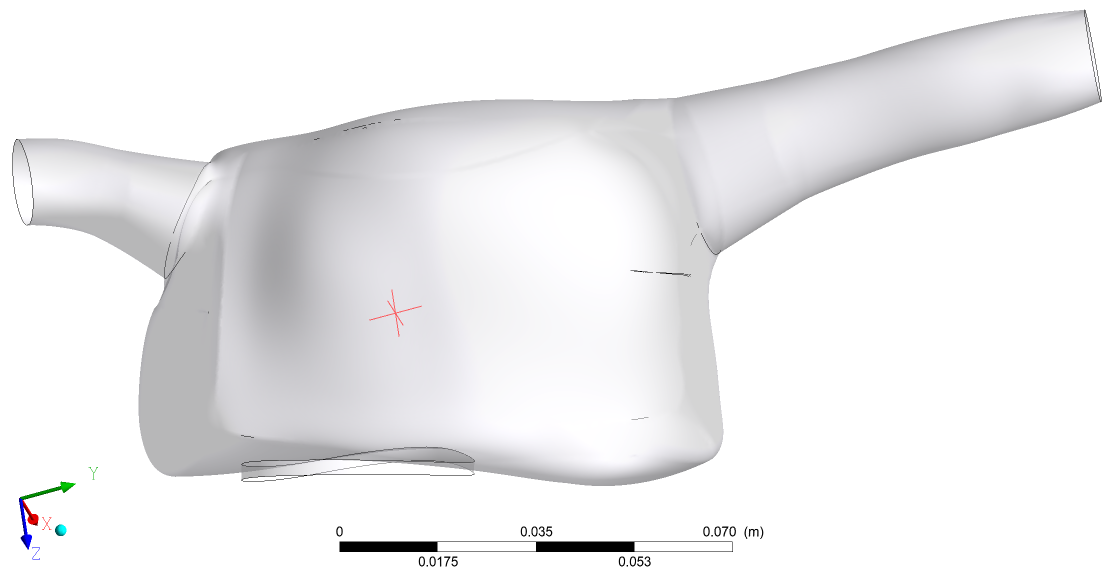


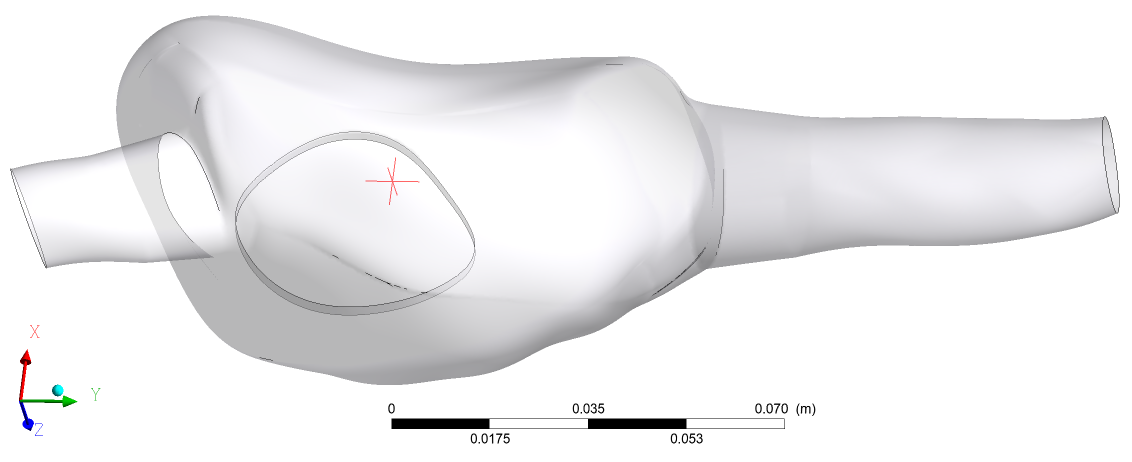

Supplement: S5 File — (DOCX) [file pone.0247438.s005.docx]
