# Supplementary material for: Computational fluid dynamics of the right atrium: Assessment of modelling criteria for the evaluation of dialysis catheters
Source: PLoS One. 2021 Feb 25;16(2):e0247438. doi: 10.1371/journal.pone.0247438 (PMC7906423; doi:10.1371/journal.pone.0247438)
Supplement: S6 File — (DOCX) [file pone.0247438.s006.docx]

**Mesh convergence analysis for the RA model:**

| Mesh nr. | Velocity [m/s] | Average WSS [Pa] | Relative error (velocity) [%] | Relative error (WSS) [%] |
| --- | --- | --- | --- | --- |
| 1 | 0.30810 | 4.75372 | 12.8253 | 1.6300 |
| 2 | 0.28194 | 4.81513 | 3.2471 | 0.3592 |
| 3 | 0.27777 | 4.82064 | 1.7182 | 0.2452 |
| 4 | 0.27786 | 4.82677 | 1.7523 | 0.1184 |
| 5 | 0.27432 | 4.83127 | 0.4545 | 0.0252 |
| 6 | 0.27308 | 4.83249 |  |  |
