# Supplementary material for: Computational fluid dynamics of the right atrium: Assessment of modelling criteria for the evaluation of dialysis catheters
Source: PLoS One. 2021 Feb 25;16(2):e0247438. doi: 10.1371/journal.pone.0247438 (PMC7906423; doi:10.1371/journal.pone.0247438)

**Comparison of Newtonian and Non-Newtonian Bird-Carreau models for vorticity and WSS quantities in the RA:**

| **Metrics** | **Average relative error [%]** | **Maximum relative error [%]** |
| --- | --- | --- |
| Vol-avg vorticity | 3.56 | 8.97 |
| Area-avg WSS | 2.16 | 5.28 |


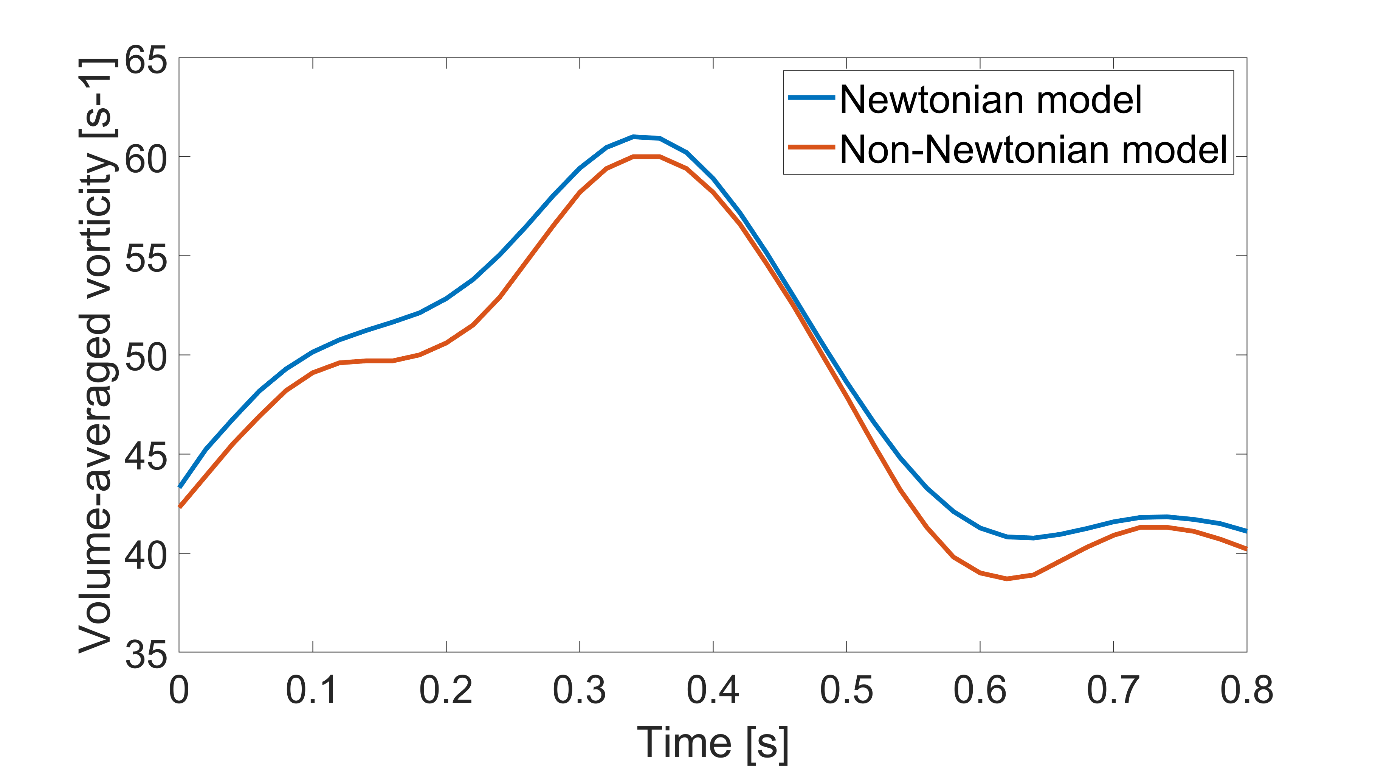


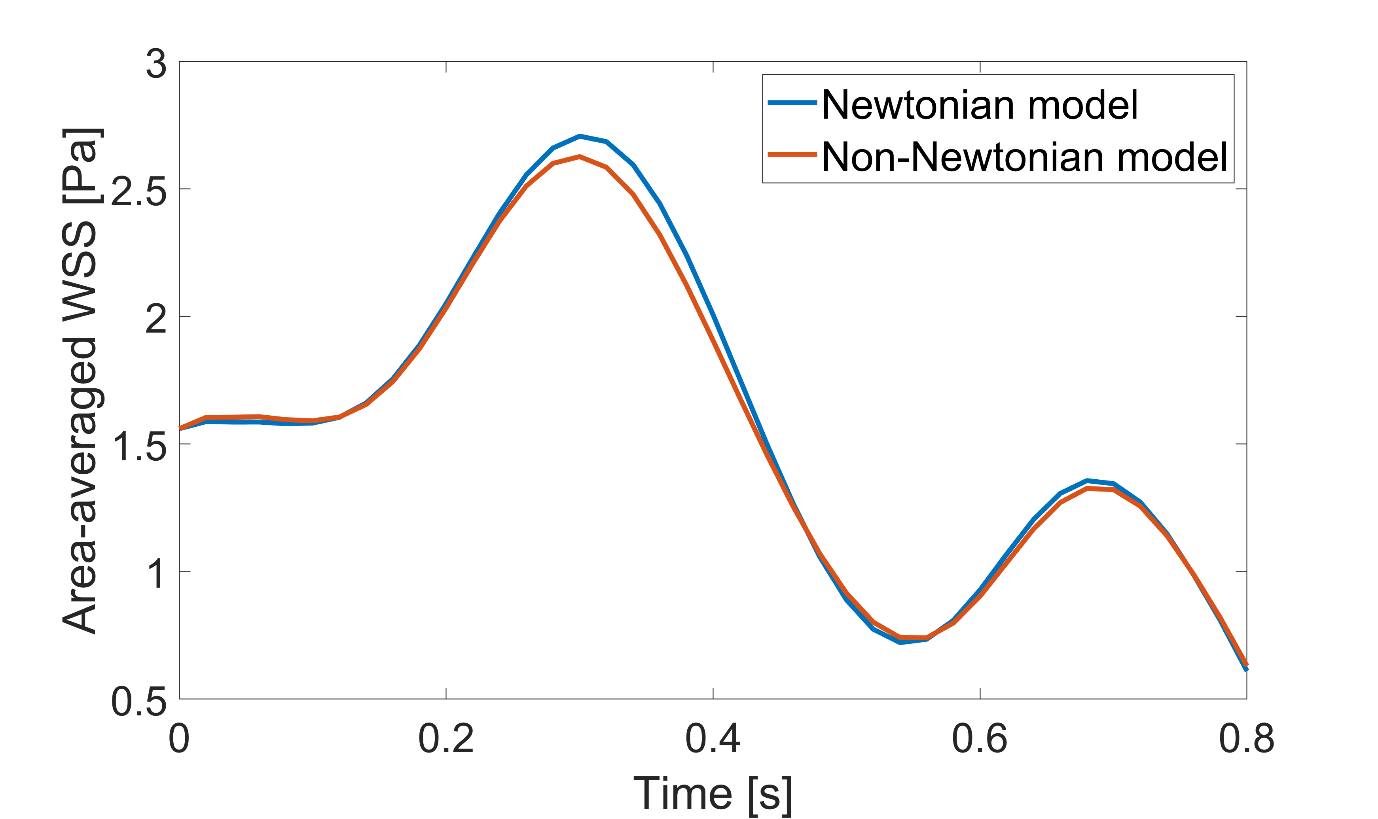

Supplement: S7 File — (DOCX) [file pone.0247438.s007.docx]
